# Supplementary material for: Time series analysis of total and direct associations between high temperatures and preterm births in Detroit, Michigan
Source: BMJ Open. 2020 Feb 5;10(2):e032476. doi: 10.1136/bmjopen-2019-032476 (PMC7045030; doi:10.1136/bmjopen-2019-032476)
Supplement: Supplementary data [file bmjopen-2019-032476supp001.pdf]

SUPPLEMENTAL MATERIAL

Table S1. Percent of preterm births attributable to 2-day mean apparent temperature (AT) on a 24.9 °C day vs. an 18.6 °C day, Detroit, MI, May-September, 1991-2001, using a case-crossover design.

Model	Time stratum	Covariates	Percent attributable	95% Confidence Interval
1	3 weeks	none	11.2	6.1, 15.9
2	2 weeks	none	11.7	6.9, 16.3
3	1 month	none	11.7	6.8, 16.4
4	3 weeks	solar radiation, wind speed, precipitation	18.7	1.3, 31.5
5	3 weeks	solar radiation, wind speed, precipitation, inverse-odds weights[s]	11.1	3.5, 17.5

[s]Inverse-odds weights calculated from the predicted odds of AT given lag day 0 and 1 of ozone, PM_{10} and NO_2.
