# Supplementary material for: Time series analysis of total and direct associations between high temperatures and preterm births in Detroit, Michigan
Source: BMJ Open. 2020 Feb 5;10(2):e032476. doi: 10.1136/bmjopen-2019-032476 (PMC7045030; doi:10.1136/bmjopen-2019-032476)
Supplement: Author's manuscript [file bmjopen-2019-032476.draft_revisions.pdf]

BMJ Open is committed to open peer review. As part of this commitment we make the peer review history of every article we publish publicly available.

When an article is published we post the peer reviewers' comments and the authors' responses online. We also post the versions of the paper that were used during peer review. These are the versions that the peer review comments apply to.

The versions of the paper that follow are the versions that were submitted during the peer review process. They are not the versions of record or the final published versions. They should not be cited or distributed as the published version of this manuscript.

BMJ Open is an open access journal and the full, final, typeset and author-corrected version of record of the manuscript is available on our site with no access controls, subscription charges or pay-per-view fees (http://bmjopen.bmj.com).

If you have any questions on BMJ Open's open peer review process please email info.bmjopen@bmj.com

BMJ Open

Total and direct associations between high temperatures and preterm births in Detroit, Michigan

Journal:	*BMJ Open*
Manuscript ID	bmjopen-2019-032476
Article Type:	Research
Date Submitted by the Author:	20-Jun-2019

Complete List of Authors:	Gronlund, Carina; University of Michigan Institute for Social Research, Survey Research Center<br>Yang, Alyssa; Urban Indian Health Institute<br>Conlon, Kathryn; University of California Davis<br>Bergmans, Rachel S.; University of Michigan Medical School,<br>Le, Hien; Chemours Co<br>Batterman, Stuart; University of Michigan, Environmental Health Sciences<br>Wahl, Robert; Michigan Department of Health and Human Services<br>Cameron, Lorraine; Michigan Department of Health and Human Services<br>O'Neill, Marie; University of Michigan, Environmental Health Sciences and Epidemiology

Keywords:	Maternal medicine < OBSTETRICS, STATISTICS & RESEARCH METHODS, EPIDEMIOLOGY

SCHOLARONE™
Manuscripts

TITLE PAGE

Title

Total and direct associations between high temperatures and preterm births in Detroit, Michigan

Authors

Carina J. Gronlund[1], Alyssa J. Yang[2], Kathryn C. Conlon[3], Rachel S. Bergmans[4], Hien Q. Le[5], Stuart A. Batterman[6], Robert L. Wahl[7], Lorraine Cameron[7], Marie S. O'Neill[6,8]

[1]University of Michigan Institute for Social Research
Survey Research Center, Social Environment and Health Program
426 Thompson St. Rm 3340
Ann Arbor, MI 48104
gronlund@umich.edu
734-615-9215

[2]Urban Indian Health Institute, Seattle, WA, USA

[3]University of California at Davis, Sacremento, CA, USA

[4]Department of Psychiatry, University of Michigan, Ann Arbor, MI, USA

[5]Health Services/Epidemiology, The Chemours Company, Wilmington, DE, USA

[6]Department of Environmental Health Sciences, University of Michigan, Ann Arbor, MI, USA

[7]Michigan Department of Health and Human Services, Lansing, MI, USA

[8]Department of Epidemiology, University of Michigan, Ann Arbor, MI, USA

Word count: 2,984

For peer review only

ABSTRACT

Background: Preterm births (PTBs) cause significant infant health risks, and several studies have found associations between high outdoor temperatures and PTB. We estimated both the total and natural direct effects (independent of particulate matter, ozone and nitrogen dioxide) of the prior two-day mean apparent temperature (AT) on PTB in the Detroit, Michigan area for the warm months (May to September), 1991-2001. We evaluated effect modification by maternal age, race, education, smoking status, and prenatal care.

Methods: We used a time series Poisson regression with splines of AT, wind speed, solar radiation, and citywide average precipitation to estimate total effects. To accommodate multiple mediators and exposure-mediator interactions, AT inverse odds weights, predicted by meteorological and air pollutant covariates, were added in a subsequent model to estimate direct effects.

Results: At 24.9 °C relative to 18.6 °C, 10.6% (95% Confidence Interval: 3.8%, 17.2%) of PTBs were attributable to the total effects of AT, and 10.4% (95% CI: 2.2%, 17.5%) to direct effects. Relative excess risks of interaction indicated that the risk of PTB with increasing temperature above 18.6 °C was significantly lower among black mothers and higher among mothers less than 19, older than 30, with late or no prenatal care, and who smoked.

Conclusion: This additional evidence of a direct association between high temperature and PTB may motivate public health interventions to reduce extreme heat exposures among pregnant women, particularly among those who may have enhanced vulnerability.

For peer review only

For peer review only - http://bmjopen.bmj.com/site/about/guidelines.xhtml

STRENGTHS AND LIMITATIONS OF THIS STUDY

- We expand on a growing literature demonstrating total effects of temperature on PTB by examining mediation of the total temperature effect by air pollutants.
- We did not have geographic identifiers for the study participants at a level as fine as residential address or indoor exposure information, and we therefore assigned time-varying environmental exposures at an outdoor, citywide level. However, a low prevalence of residential air conditioning (32%) in the study time period suggests that outdoor exposures may have more accurately reflected indoor exposures than in more recent studies.
- We account for multiple mediators and potential exposure-mediator interactions by applying a straightforward technique—the use of inverse odds weights—to separate the total effects of temperature on PTB from the direct effects.
- We used methods examining potential synergistic effects, as opposed to just examining statistical interactions, by other characteristics that confer vulnerability to PTB.

For peer review only

For peer review only - http://bmjopen.bmj.com/site/about/guidelines.xhtml

INTRODUCTION

Preterm births (PTBs) are defined as births that occur before 37 gestational weeks. Babies born prematurely are at greater risk of infant death and impaired health, such as cerebral palsy and impairments of cognitive development, hearing, vision, respiration, and digestion. In 2016, 10% of babies were born prematurely, with statewide rates ranging 7.8-13.7%[1]. Within-state disparities in preterm birth rates can be even greater: from 1990-2010, 18% of births in Detroit, Michigan were preterm births[2].

Certain risk factors are known to be associated with PTB, such as cigarette smoking, alcohol use, hypertension, and diabetes[1]. Air pollution is associated with PTB[3 4], and higher ambient temperature has been proposed as a risk factor for PTB[5 6].

High ambient temperature could pose a risk for PTB through several biological pathways. Higher temperatures may increase stress levels[7]. Stress increases the levels of cortisol and epinephrine, potentially leading to the secretion of placental corticotropin-releasing hormones (CRH). Placental CRH can then activate the fetal hypothalamic-pituitary-adrenal (HPA) axis, which could prompt the fetal expression of cortisol and dehydroepiandrosterone-sulfate (DHEA-S) and placental release of estriol and prostaglandins, potentially triggering PTB[8]. Dehydration due to high temperatures and sweating could also reduce blood flow to the uterus and induce a greater release of antidiuretic hormone and oxytocin, which could trigger labor onset[9].

Several studies have evaluated possible associations between high ambient temperature and PTB, with mixed results[5 6]. Studies in cities in Australia, Quebec, China, Belgium, Italy, Spain and across the U.S. have all found significant positive associations between hot temperatures within the week preceding delivery and PTB[5 6 10-18].

However, earlier, rigorously conducted time series analyses of temperature and PTB in Germany and in London, England did not find associations[19 20]. Likewise, in Chicago, Illinois,

For peer review only

Porter et al. found no effect of the July 1995 heat wave on gestational length[21]. Sources of heterogeneity among these effect estimates may include differences in study design, prevailing climate and regional adaptation, population structure, exposure assessment, critical windows of exposure considered, and methods of gestational age assessment[14]. Additionally, studies often control for air pollution exposure, although the argument has been made that treating air pollutants as confounders of temperature-health associations is inappropriate given that high temperatures can contribute to increased air pollutant concentrations, and both temperature and air pollution are affected by sunlight[22]. These differences in the manner in which air pollutants are accounted for in the temperature-PTB modeling may also account for the heterogeneity observed in this literature.

We investigated the association between high AT and PTB in the Detroit, Michigan area, estimating both the total effects of temperature as well as the natural direct effects, excluding potential mediation effects by the air pollutants ozone, particulate matter with an aerodynamic diameter of less than 10 micrometers (PM_{10}), and nitrogen dioxide (NO_2). We further examined whether the maternal risk factors of age, race, education, smoking, and level of prenatal care modified the association between high AT and PTB.

METHODS

Outcome Variable

Birth outcome data were derived from birth certificate records kept by the Michigan Department of Health and Human Services[4]. This sample population included live, singleton births that occurred from January 1, 1991 to December 31, 2001 in ZIP codes within 4 km of one of three air quality monitors (the Allen Park, Linwood, and East 7 Mile monitors) in and bordering Detroit, Michigan[4]. These births were further restricted to only PTBs, defined as births that were less than 37 gestational weeks, and births that only occurred May-September, in order

For peer review only - http://bmjopen.bmj.com/site/about/guidelines.xhtml

to focus on heat exposures. Birth certificate data also included date of birth and maternal age, race, smoking status, education level, and level of prenatal care.

Identification of Confounders and Mediators

A directed acyclic graph (DAG) was constructed to define the causal framework and elucidate potential mediating effects of air pollutants in the association between AT and PTB (Figure 1). Primary and secondary air pollutants can be considered mediators of the temperature-PTB association given that: 1) they have been associated with the health outcome[3], 2) high temperature enhances the rate of ground-level ozone formation, and 3) power plant emissions increase when temperatures rise to accommodate additional demand for air conditioning[23]. Furthermore, the weather system is a causal parent to both AT and the pollutants given that meteorological conditions such as wind speed, precipitation, and cloud cover affect pollutant formation and exposure as well as sunlight, which increases AT and promotes ozone formation[24].

Exposure Variables

Daily mean temperature, dew point temperature, and wind speed were obtained from the National Center for Environmental Information[25]. Data from the Detroit City Airport was used due to its proximity to the mothers' ZIP codes of residence. AT was calculated using the following formula: [-2.653 + (0.994*Temperature in °C) + 0.0153*(Dew Point Temperature in °C)^2][26]. From Detroit City Airport, 11% and 15% of temperatures and dew points were missing, respectively, so data from Detroit Metropolitan Airport were used to replace these missing data. Among the non-missing values, daily AT from Detroit City Airport was highly correlated with that from Detroit Metropolitan Airport (Pearson's correlation coefficient = 0.98).

The total amount of direct and diffuse solar radiation received on a horizontal surface during each 60-minute period at the Detroit City Airport was retrieved from the National Solar Radiation Database[27]. These data were modeled from meteorological data including cloud cover,

For peer review only - http://bmjopen.bmj.com/site/about/guidelines.xhtml

aerosol, and ozone data from sources such as sun photometers and satellites and albedo data[28]. We further estimated daily means from the hourly solar radiation values.

Daily precipitation totals were obtained from Oregon State University's PRISM Climate Group[29]. These are modeled at a 4-km resolution based on observations and a climatologically-aided interpolation process. Rasters were cropped to the City of Detroit and daily citywide averages were calculated.

Daily eight-hour maximum ozone and daily mean NO_2 and PM_{10} concentrations were obtained from the Environmental Protection Agency for all Wayne County, Michigan monitors and averaged by day and pollutant[30]. Only daily monitor values for which 18 of the 24 hourly values were available were retained, resulting in substantial missingness, which was addressed in the statistical analysis below.

Patient and Public Involvement

Patients were not involved.

Statistical Analysis

Case-crossover analysis is commonly used in studies of PTB and temperature[14 15 31]. Because PTB is not a rare event in this particular population, the case-crossover odds ratios would not approximate risk ratios. Therefore, we used a time series design with Poisson regression, controlling for seasonal effects with a cubic b-spline for day-of-year, with 5, 2 or 8 knots, and a cubic b-spline for year with 2 knots. For AT and the covariates, non-linearity was considered by initially modeling each as a b-spline with three knots and selecting a single knot for subsequent modeling of the covariate as a piecewise linear spline when substantial nonlinearity was visually evident. The 95% confidence intervals (CIs) were constructed from the 2.5th and 97.5th percentiles of 500 bootstrapped samples. In sensitivity analyses, given its wide

For peer review only - http://bmjopen.bmj.com/site/about/guidelines.xhtml

usage for this research question, a time-stratified case-crossover design was used with time-strata defined as two- or three-week periods.

To account for missing air pollutant values, we conducted multiple imputations using chained equations. We used a more general model including lag days 0-2 of the above meteorological and pollutant values, generating three imputations following two burn-ins. All subsequent analyses were conducted on each of the three resulting data sets.

To estimate the total effects of AT, we included terms for solar radiation, wind speed, and precipitation in the Poisson model, which blocked any of the paths in the DAG from AT to PTB that did not pass through the mediators (Figure 1). However, to estimate natural direct effects, we used a more generalizable technique—inverse odds weighting—given that we had multiple mediators and as well as potential exposure-mediator interactions[32]. In this technique, we first fit a standard linear regression of two-day mean AT (meanAT01) on the air pollutant mediators and covariates (solar radiation, wind speed, precipitation). We then estimated:

$$\text{inverse odds} = 1 / \exp(\text{predicted meanAT01} / \sigma^2) \qquad \text{Equation 1}$$

where σ^2 was the model mean squared-error. The inverse odds were then used as weights in the subsequent analysis of PTB and AT with the other covariates but not the air pollutants. The weights render AT independent of the mediators, thereby allowing the estimation of AT separately from the effects of the air pollutant mediators on PTB. Total and direct effects were calculated from each of the three imputed data sets and then each averaged.

To assess the public health impact of AT on PTB, we estimated the attributable fraction (AF), or the percent of PTB attributable to high AT among women exposed to the high AT. The AF was estimated as: 1 / (1 – RR) * 100% where the RR was for the 95th percentile (24.9 °C) relative to the 50th percentile (18.6 °C) of May-September two-day mean AT. Indirect effects

For peer review only - http://bmjopen.bmj.com/site/about/guidelines.xhtml

were estimated as the difference between the total effects and the direct effects. The 95% confidence intervals (CIs) were constructed from the 2.5th and 97.5th percentiles of 500 bootstrapped samples.

To understand how the effects varied among maternal subgroups, we simultaneously included interaction terms between the AT spline and the indicator variables for black race, age 16-19, age 30 years or older, low prenatal care (late or no prenatal care), current smoker, and no high school education. We expanded the data set such that we had rows for each unique combination of date and daily exposures, race, age group, education, prenatal care, and smoking status. Given the large number of zero counts, we specified a negative binomial distribution rather than a Poisson distribution. For public health significance, we were interested in the absolute rather than the relative increase in PTB risk due to synergistic effects between temperature and each modifier of interest. Therefore we focused on additive, rather than the multiplicative, interactions. Relative excess risk due to interaction (RERI) [33] was calculated for each potential modifier as:

$$RR_{AT=95th,EM=1} - RR_{AT=95th,EM=0} - RR_{AT=50th,EM=1} + 1 \qquad \text{Equation 2}$$

where EM was the effect modifier of interest and the denominator of each RR was the risk at the 50th percentile of AT (18.6 °C) and the absence of the modifier (EM = 0). RERI confidence CIs were bootstrapped from 1,000 samples. All analyses were conducted in SAS 9.4 (Copyright 2016, SAS Institute Inc., Cary, NC, USA) using PROC GLIMMIX, which allows for multiple splines in a Poisson regression.

RESULTS

There were 9,053 singleton preterm births in this Detroit-area sample, May-September, 1991-2001. Of these, 27.0% were among white mothers and 71.1% were among black mothers.

For peer review only - http://bmjopen.bmj.com/site/about/guidelines.xhtml

A majority of the mothers were non-smokers (73.1%), had prenatal care (55.6%), were 20-29 years of age (53.2%) and had at least a high school education (60.2%) (Table 1).

On average, AT exposure was 18.0 °C, and among the days when AT was greater than the time-period-specific daily median of 18.6 °C (i.e., for the upper end of the temperature spline), AT averaged 1.5 °C higher. A total of 53% of the cases occurred on days with no rain, so the geometric mean precipitation was 0.4 mm. The mean ozone concentration was 44.8 ppb and the maximum was 102 ppb (Table 2). In examining the daily time series of ozone over the study period, the three-year running averages of the fourth highest daily 8-hour maximum value of ozone ranged from 77 to 92 ppb, suggesting that the (current) National Ambient Air Quality Standards (70 ppb) had been exceeded in each year of the study.

To address potential sensitivity of the results to control for season, we varied the df in the day-of-year term, using 2, 5, and 8 df. The results were not highly sensitive to this choice, with the point estimates of the percentages of PTB attributable to AT ranging only from 8.1% (8 df) to 9.9% (2 df) among pregnant women exposed to days when AT was 24.9 °C vs exposure on 18.6 °C days (Table 3). In case-crossover analyses, the resulting odds ratios were also statistically significantly greater than 1.0, regardless of whether the time strata were two-week, three-week, or one-month periods. However, the odds ratios were greater in magnitude than the risk ratios estimated in the time series design, which is expected, given that odds ratios overestimate risk ratios when events are not rare.

The total effects were estimated as 10.6% (95% CI: 3.8%, 17.2%) of PTBs attributable to AT among pregnant women exposed to days when AT was 24.9 °C vs. exposure on 18.6 °C days. Accounting for mediation of this effect by PM_{10}, ozone, and NO_2 by using inverse odds weights, the direct effect of AT on preterm birth was decreased to 10.4% (95% CI: 2.2%,

For peer review only - http://bmjopen.bmj.com/site/about/guidelines.xhtml

17.5%), although the difference between the two values was not statistically significant (indirect effect = 0.3%, 95% CI: -1.7%, 2.6%).

In examining the RERI from interactions between AT and maternal characteristics, black race was found to be protective, with an RERI significantly less than 0 (Table 4). The magnitudes of RERIs are not meaningful, but this result indicates that the association between high temperature and PTB is weaker among women of black race than non-black race. In contrast, RERIs for age 16-19 years, age > 30 years, low prenatal care and tobacco smoking were all significantly greater than 0, indicating that the association between PTB and high temperature is stronger among women with these characteristics.

DISCUSSION

The strong association between short-term temperature exposure and PTB in Detroit, Michigan was consistent with several other studies' observed associations between PTB or diminished gestational age and temperature exposures within the week prior to delivery at regionally high temperatures. Regions in which these associations have been found include Central Australia (8.3% at 40 °C daily maximum temperature)[13], Northern California, USA (11.6% increase for a 5.6 °C increase in weekly average AT in the warm season)[31], Alabama, USA (32.4% increase with two consecutive days of daily mean temperatures above the 98th percentile)[34], Barcelona, Spain (5-day reduction in average gestational age with heat index above the 99th percentile)[10], Rome, Italy (1.9% increase per 1 °C increase in maximum AT in the prior two days and 19% increase during heat waves)[35], Brisbane, Australia (13%-100% increase during heat waves), in an aggregated sample of 12 U.S. cities (12-16% increase with 2.8 °C increase in prior week)[14], and Southern China (7% increase with previous-week temperatures above 95th percentile)[12].

For peer review only - http://bmjopen.bmj.com/site/about/guidelines.xhtml

One strength of our study may be a tighter correspondence between the true ambient temperatures experienced by our sample and the measured outdoor ambient AT given the low air conditioning prevalence in the Detroit area during the time period. Specifically, in 1993, the last year for which county-specific American Housing Survey data were available in the Detroit area, only 32% of Wayne County households had central air conditioning[36]. Furthermore, central air conditioning prevalence was low in this region despite a "hot-summer humid continental climate" Köppen Climate Classification[37], allowing a fairly wide range of warm season AT exposure over which AT-PTB associations could be examined. In contrast, regions where associations between temperature and PTB were null in rigorously conducted daily time series studies included Brandenburg and Saxony, Germany[20] and London, UK[19], which are in "temperate oceanic climates," where all months have average temperatures below 22 °C [37]. Additionally, Guo et al.[12] found an association between previous-week temperature and PTB only in the "hot" region of China, defined as having annual average temperatures 19 °C or higher. Furthermore, in a survival analysis of previous-week temperature and PTB in 18 European cities, the pooled effect estimates were null, and no individual city results were presented[38]. Again, with the exception of 4 cities, these European cities were all in cooler climates where all months have average temperatures below 22 °C[37]. This suggests that in regions where the threat of extremely high temperatures is rare, PTB is not triggered at warm temperatures, regardless of relative temperature thresholds, even at the same absolute temperatures at which heat-associated PTB is experienced in warmer climates. This could be due to differences in emotional stress[39] or physiological stress responses to warm temperatures between climates. Alternatively, the heterogeneity in effect estimates could be due to regional differences in the misclassification of the true individually-experienced temperatures by outdoor

For peer review only - http://bmjopen.bmj.com/site/about/guidelines.xhtml

temperatures. Finally, in the 18-city European study, the PTB rate was only 5%, suggesting that PTB etiologies may differ between Europe and the U.S., in which case the pregnancies in the European cohorts could have been less susceptible physiologically to high temperature[38].

Other strengths of our study include our consideration of near-surface solar radiation and weather conditions as confounders of the total effects of AT, air pollutants as mediators rather than confounders, and estimation of the direct effects of AT as distinct from those mediated by air pollutants. By using an analysis technique that was “agnostic” to exposure-mediator interactions and could accommodate multiple mediators[32], we found that most if not all of the effect of AT on PTB is direct, and not mediated by daily changes in air pollution concentrations. Furthermore, estimating and reporting RERIs, rather than merely reporting statistical interactions in the models, provided evidence for synergistic effects of high temperature with the independent risk factors for PTB of prenatal care, smoking status, and age. Interestingly, in this majority-black population, the risk of PTB with high temperatures was actually lower among black mothers, after controlling for age, education, smoking status, and prenatal care.

Limitations include the fact that the study design does not distinguish, subtypes of preterm birth based on gestational duration or other classification (e.g. spontaneous versus medically indicated) or provide information on how much earlier the birth was, e.g., a one-day premature birth vs. a six-week premature birth. The model used assumes that the baseline PTB rate does not change over time other than what is being captured with a spline for year and spline for day-of-year. However, varying the degrees of freedom in the day-of-year term from 2 to 8, with mild effect. Additionally, the prevalence of characteristics enhancing vulnerability to PTB may have changed since the study period, thereby decreasing the generalizability of these results to the present-day population of this region. Future research

For peer review only - http://bmjopen.bmj.com/site/about/guidelines.xhtml

should employ present-day cohorts of pregnant women linked with refined temperature exposure measurements, including indoor and neighborhood temperature exposure estimates. These research refinements will better characterize the thermal exposures and severity of heat-induced PTB, or more specifically, gestational length, and better identify which pregnancies are particularly vulnerable to early parturition on hot days.

Despite the aforementioned limitations, given the evidence from this and other studies, pregnant women, in addition to older adults, should be considered as a group vulnerable to short-term heat health effects when considering housing and climate adaptation measures in Detroit and similar or warmer climates.

For peer review only

CONTRIBUTORSHIP STATEMENT

MSO, SAB, HQL, RLW, and LC obtained the data or resources, critiqued the analysis, and reviewed the manuscript. RSB and KCC assisted with preliminary analyses, critiqued the analysis and reviewed the manuscript. AJY performed and drafted preliminary analyses and reviewed the manuscript. CJG directed preliminary analyses, revised the analysis, and revised the manuscript. We thank Leah Comment for early-stage data management and analysis assistance. We also thank Patricia Maina for her contribution to the preterm birth and heat literature review and Sung Kyun Park for early stage advice.

FUNDING

This work was supported by a Michigan Institute for Clinical and Health Research Postdoctoral Translational Scholars Fellowship (2UL1TR000433-06), National Institute of Environmental Health Sciences grants K99ES026198 and P30ES017885, Cooperative Agreement Number EH001124 from the Centers for Disease Control and Prevention (CDC), National Science Foundation grant 1520803, and CDC/ National Institute for Occupational Safety and Health grant T42 OH008455. Its contents are solely the responsibility of the authors and do not necessarily represent the official views of the CDC or the Michigan Department of Health and Human Services. None of the funders participated in the design, collection, analysis, or interpretation of the data.

COMPETING INTERESTS

None declared.

DATA SHARING STATEMENT

Birth outcome data were derived from birth certificate records kept by the Michigan Department of Health and Human Services (MDHHS).[4] These confidential data may be obtained from the MDHHS Division for Vital Records and Health Statistics following completion of a data use agreement. The exposure data are publicly available and were obtained as described in the Methods. The processed exposure data may be requested from C. Gronlund (gronlund@umich.edu).

For peer review only

REFERENCES

1. Centers for Disease Control and Prevention (CDC). Premature Birth 2017 [updated 11/6/2017. Available from: https://www.cdc.gov/features/prematurebirth/ accessed Jan 29 2018.
2. Make Your Date Detroit, Center for Advanced Obstetrical Care and Research, Wayne State University. Why This Matters--Facts 2017 [Available from: https://makeyourdate.org/facts/ accessed Jan 29 2018.
3. Sun XL, Luo XP, Zhao CM, et al. The association between fine particulate matter exposure during pregnancy and preterm birth: a meta-analysis. *BMC Pregnancy Childbirth* 2015;15:12. doi: 10.1186/s12884-015-0738-2
4. Le HQ, Batterman SA, Wirth JJ, et al. Air pollutant exposure and preterm and term small-for-gestational-age births in Detroit, Michigan: Long-term trends and associations. *Environment International* 2012;44:7-17. doi: http://dx.doi.org/10.1016/j.envint.2012.01.003
5. Carolan-Olah M, Frankowska D. High environmental temperature and preterm birth: a review of the evidence. *Midwifery* 2014;30(1):50-9. doi: 10.1016/j.midw.2013.01.011 [published Online First: 2013/03/12]
6. Strand LB, Barnett AG, Tong S. The influence of season and ambient temperature on birth outcomes: a review of the epidemiological literature. *Environ Res* 2011;111(3):451-62. doi: 10.1016/j.envres.2011.01.023 [published Online First: 2011/02/22]
7. McMorris T, Swain J, Smith M, et al. Heat stress, plasma concentrations of adrenaline, noradrenaline, 5-hydroxytryptamine and cortisol, mood state and cognitive performance. *International Journal of Psychophysiology* 2006;61(2):204-15. doi: https://doi.org/10.1016/j.ijpsycho.2005.10.002
8. Behrman RE, Adashi EY, Allen MC, et al. Preterm Birth: Causes, Consequences, and Prevention. In: Medicine Io, ed. Report Brief. Washington, DC: Institute of Medicine, 2006.
9. Stan C, Boulvain M, Hirsbrunner-Amagbaly P, et al. Hydration for treatment of preterm labour. *Cochrane Database Syst Rev* 2002;2
10. Dadvand P, Basagana X, Sartini C, et al. Climate extremes and the length of gestation. *Environ Health Perspect* 2011;119(10):1449-53. doi: 10.1289/ehp.1003241 [published Online First: 2011/06/11]
11. Strand LB, Barnett AG, Tong S. Maternal exposure to ambient temperature and the risks of preterm birth and stillbirth in Brisbane, Australia. *Am J Epidemiol* 2012;175(2):99-107. doi: 10.1093/aje/kwr404 [published Online First: 2011/12/15]
12. Guo TJ, Wang YY, Zhang HG, et al. The association between ambient temperature and the risk of preterm birth in China. *Sci Total Environ* 2018;613:439-46. doi: 10.1016/j.scitotenv.2017.09.104
13. Mathew S, Mathur D, Chang AB, et al. Examining the Effects of Ambient Temperature on Pre-Term Birth in Central Australia. *International Journal of Environmental Research and Public Health* 2017;14(2) doi: 10.3390/ijerph14020147
14. Ha SD, Liu DP, Zhu YY, et al. Ambient Temperature and Early Delivery of Singleton Pregnancies. *Environmental Health Perspectives* 2017;125(3):453-59. doi: 10.1289/ehp97
15. Basu R, Chen H, Li DK, et al. The impact of maternal factors on the association between temperature and preterm delivery. *Environmental Research* 2017;154:109-14. doi: 10.1016/j.envres.2016.12.017

For peer review only - http://bmjopen.bmj.com/site/about/guidelines.xhtml

16. Schifano P, Asta F, Dadvand P, et al. Heat and air pollution exposure as triggers of delivery: A survival analysis of population-based pregnancy cohorts in Rome and Barcelona. *Environment International* 2016;88:153-59. doi: 10.1016/j.envint.2015.12.013
17. He JR, Liu Y, Xia XY, et al. Ambient Temperature and the Risk of Preterm Birth in Guangzhou, China (2001-2011). *Environ Health Perspect* 2015 doi: 10.1289/ehp.1509778 [published Online First: 2015/12/17]
18. Arroyo V, Diaz J, Ortiz C, et al. Short term effect of air pollution, noise and heat waves on preterm births in Madrid (Spain). *Environmental Research* 2016;145:162-68. doi: 10.1016/j.envres.2015.11.034
19. Lee SJ, Hajat S, Steer PJ, et al. A time-series analysis of any short-term effects of meteorological and air pollution factors on preterm births in London, UK. *Environ Res* 2008;106(2):185-94. doi: 10.1016/j.envres.2007.10.003 [published Online First: 2007/11/21]
20. Wolf J, Armstrong B. The association of season and temperature with adverse pregnancy outcome in two German states, a time-series analysis. *PLoS One* 2012;7(7):e40228. doi: 10.1371/journal.pone.0040228 [published Online First: 2012/07/14]
21. Porter KR, Thomas SD, Whitman S. The relation of gestation length to short-term heat stress. *American Journal of Public Health* 1999;89(7):1090-92. doi: 10.2105/ajph.89.7.1090
22. Buckley JP, Samet JM, Richardson DB. Commentary: Does air pollution confound studies of temperature? *Epidemiology* 2014;25(2):242-5. doi: 10.1097/ede.0000000000000051 [published Online First: 2014/02/04]
23. Abel D, Holloway T, Kladar RM, et al. Response of Power Plant Emissions to Ambient Temperature in the Eastern United States. *Environ Sci Technol* 2017;51(10):5838-46. doi: 10.1021/acs.est.6b06201
24. Environmental Protection Agency (EPA). Ozone Pollution and Your Patient's Health: What is Ozone? 2018 [Available from: https://www.epa.gov/ozone-pollution-and-your-patients-health/what-ozone accessed December 20 2018.
25. National Climatic Data Center. Integrated Surface Database Lite 2010 [Available from: http://www.ncdc.noaa.gov/oa/climate/isd/index.php accessed July 2010.
26. Oswald EM, Rood RB, Zhang K, et al. An investigation into the spatial variability of near-surface air temperatures in the Detroit, MI metropolitan region. *J Appl Meteorol Climatol* 2012;51(7):1290-304. doi: 10.1175/JAMC-D-11-0127.1
27. National Solar Radiation Database, 1991-2010 Update [Available from: https://rredc.nrel.gov/solar/old_data/nsrdb/ accessed August 25 2017.
28. Wilcox S. National Solar Radiation Database 1991-2010 Update: User's Manual. In: National Renewable Energy Laboratory, ed.: U.S. Department of Energy, Office of Energy Efficiency & Renewable Energy, 2012.
29. PRISM Climate Group. PRISM Climate Data 2016 [Available from: http://www.prism.oregonstate.edu/ accessed March 6 2016.
30. U.S. Environmental Protection Agency. AirData 2015 [Available from: http://aqsdr1.epa.gov/aqsweb/aqstmp/airdata/download_files.html accessed Feb 5 2015.
31. Avalos LA, Chen H, Li DK, et al. The impact of high apparent temperature on spontaneous preterm delivery: a case-crossover study. *Environmental Health* 2017;16 doi: 10.1186/s12940-017-0209-5

For peer review only - http://bmjopen.bmj.com/site/about/guidelines.xhtml

32. Nguyen QC, Osypuk TL, Schmidt NM, et al. Practical Guidance for Conducting Mediation Analysis With Multiple Mediators Using Inverse Odds Ratio Weighting. *American Journal of Epidemiology* 2015;181(5):349-56. doi: 10.1093/aje/kwu278
33. Nie L, Chu H, Li F, et al. Relative Excess Risk Due to Interaction: Resampling-based Confidence Intervals. *Epidemiology* 2010;21(4):552-56.
34. Kent ST, McClure LA, Zaitchik BF, et al. Heat Waves and Health Outcomes in Alabama (USA): The Importance of Heat Wave Definition. *Environ Health Perspect* 2013 doi: 10.1289/ehp.1307262 [published Online First: 2013/11/26]
35. Schifano P, Lallo A, Asta F, et al. Effect of ambient temperature and air pollutants on the risk of preterm birth, Rome 2001-2010. *Environ Int* 2013;61:77-87. doi: 10.1016/j.envint.2013.09.005 [published Online First: 2013/10/10]
36. United States. Bureau of the Census. American Housing Survey, 1993: MSA Core and Supplement File: [distributor], 2006.
37. Peel MC, Finlayson BL, McMahon TA. Updated world map of the Köppen-Geiger climate classification. *Hydrol Earth Syst Sci* 2007;11(5):1633-44. doi: 10.5194/hess-11-1633-2007
38. Giorgis-Allemand L, Pedersen M, Bernard C, et al. The Influence of Meteorological Factors and Atmospheric Pollutants on the Risk of Preterm Birth. *American Journal of Epidemiology* 2017;185(4):247-58. doi: 10.1093/aje/kww141
39. Lin YF, Hu WJ, Xu J, et al. Association between temperature and maternal stress during pregnancy. *Environmental Research* 2017;158:421-30. doi: 10.1016/j.envres.2017.06.034

For peer review only

For peer review only - http://bmjopen.bmj.com/site/about/guidelines.xhtml

TABLES

Table 1. Demographics of Preterm Births in Detroit, MI Study Area, May-September, 1991-2001.

	N	%
Total	9,053	100
Maternal Race		
White	2,443	27.0
Black	6,433	71.1
Other	177	1.9
Maternal Smoking Status		
Non-Smoker	6,621	73.1
Smoker	2,345	25.9
Missing	87	1.0
Level of Prenatal Care		
Prenatal Care	5,037	55.6
Late or No Prenatal Care	2,960	32.7
Missing	1,056	11.7
Maternal Age		
16-19 years	1,782	19.7
20-29 years	4,821	53.2
≥ 30 years	2,450	27.1
Maternal Education		
Less than High School	3,485	38.5
High School or Higher	5,451	60.2
Missing	117	1.3

Table 2. Daily Exposures Among the PTB Cases, Detroit, MI Area, May-September, 1991-2001.

	N	Median	Mean	Min	Max
Two-day mean apparent temperature, °C	9,053	18.6	18.0	3.1	28.0
Two-day mean apparent temperature 18.6 °C and above	9,053	0.1	1.5	0.0	9.4
Mean solar radiation (W/m^2)	9,053	226.4	217.7	38.1	352.8
Total precipitation (mm)[a]	9,053	0.0	0.4	0.0	56.7
Mean wind speed (m/s)	9,053	3.6	3.6	0.1	8.0
Maximum 8-hour average ozone (ppb)	9,039	43.0	44.8	3.2	102.0
Mean particulate matter, 10 µm or less ($\mu g/m^3$)[a]	8,331	39.0	38.5	8.0	158.0
Mean nitrogen dioxide (ppb)[a]	8,956	18.4	19.7	0.0	72.8

[a] Geometric means are provided. Values were natural-log-transformed in the regression analyses.

For peer review only - http://bmjopen.bmj.com/site/about/guidelines.xhtml

Table 3. Relative Risk of Preterm Birth and Percent of Preterm Births Attributable to 2-day Mean Apparent Temperature (AT) on 24.9 °C days vs. 18.6 °C days, Detroit, MI Area, May-September, 1991-2001.

Model	Knots in day-of-year spline	Covariates	Relative Risk (95% Confidence Interval)	Percent attributable (95% Confidence Interval)
1	2	none	1.11 (1.02, 1.09)	9.9 (2.4, 16.4)
2	5	none	1.11 (1.03, 1.20)	9.8 (2.6, 16.5)
3	8	none	1.09 (1.01, 1.18)	8.1 (1.0, 17.6)
4	5	solar radiation, wind speed, precipitation	1.12 (1.04, 1.21)	10.6 (3.8, 17.2)
5	5	solar radiation, wind speed, precipitation, inverse-odds weights[s]	1.12 (1.02, 1.21)	10.4 (2.2, 17.5)

[s]Inverse-odds weights calculated from the predicted odds of AT given lag day 0 and 1 of ozone, PM_{10} and NO_2.

Table 4. Relative Excess Risk Due to Interaction (RERI) for Interactions of 2-Day Mean Apparent Temperature (AT) on 24.9 °C days vs. 18.6 °C Days with Maternal Characteristics, Detroit, MI Area, May-September, 1991-2001.

Characteristic	RERI	95% Confidence Interval
Black race	-1.5	-1.9, -1.0
Age 16-19 years	0.50	0.22, 0.76
Age > 30 years	0.34	0.08, 0.65
Low prenatal care	0.44	0.19, 0.65
No high school	0.19	-0.06, 0.43
Smoker	0.52	0.29, 0.76

With 5 knots in the day-of-year spline.

For peer review only - http://bmjopen.bmj.com/site/about/guidelines.xhtml

FIGURE LEGENDS

Figure 1. Directed Acyclic Graph of the Causal Framework for Mediation of the Association Between Apparent Temperature (AT) and Preterm Birth (PTB) by Air Pollutants.

For peer review only

For peer review only - http://bmjopen.bmj.com/site/about/guidelines.xhtml

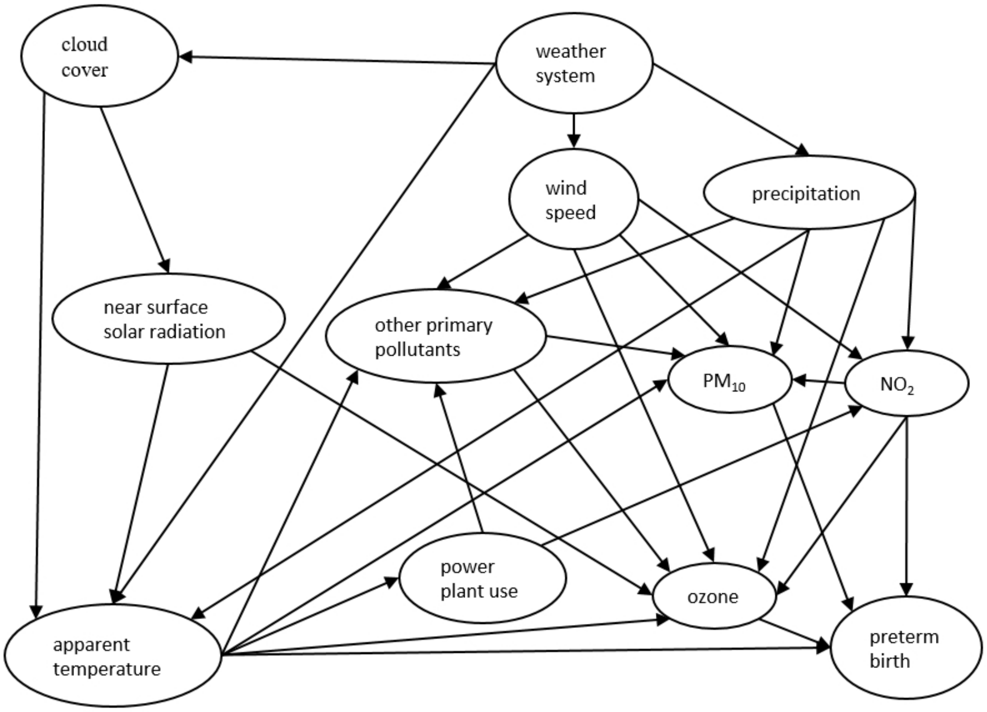

PM_{10} = particulate matter less than 10 microns in aerodynamic diameter

NO_2 = nitrogen dioxide

BMJ Open

Total and direct associations between high temperatures and preterm births in Detroit, Michigan

Journal:	*BMJ Open*
Manuscript ID	bmjopen-2019-032476.R1
Article Type:	Original research
Date Submitted by the Author:	14-Nov-2019
Complete List of Authors:	Gronlund, Carina; University of Michigan Institute for Social Research, Survey Research Center<br>Yang, Alyssa; Urban Indian Health Institute<br>Conlon, Kathryn; University of California Davis<br>Bergmans, Rachel S.; University of Michigan Medical School,<br>Le, Hien; Chemours Co<br>Batterman, Stuart; University of Michigan, Environmental Health Sciences<br>Wahl, Robert; Michigan Department of Health and Human Services<br>Cameron, Lorraine; Michigan Department of Health and Human Services<br>O'Neill, Marie; University of Michigan, Environmental Health Sciences and Epidemiology
**Primary Subject Heading**:	Epidemiology
Secondary Subject Heading:	Public health
Keywords:	Maternal medicine < OBSTETRICS, STATISTICS & RESEARCH METHODS, EPIDEMIOLOGY

SCHOLARONE™ Manuscripts

I, the Submitting Author has the right to grant and does grant on behalf of all authors of the Work (as defined in the below author licence), an exclusive licence and/or a non-exclusive licence for contributions from authors who are: i) UK Crown employees; ii) where BMJ has agreed a CC-BY licence shall apply, and/or iii) in accordance with the terms applicable for US Federal Government officers or employees acting as part of their official duties; on a worldwide, perpetual, irrevocable, royalty-free basis to BMJ Publishing Group Ltd ("BMJ") its licensees and where the relevant Journal is co-owned by BMJ to the co-owners of the Journal, to publish the Work in this journal and any other BMJ products and to exploit all rights, as set out in our licence.

The Submitting Author accepts and understands that any supply made under these terms is made by BMJ to the Submitting Author unless you are acting as an employee on behalf of your employer or a postgraduate student of an affiliated institution which is paying any applicable article publishing charge ("APC") for Open Access articles. Where the Submitting Author wishes to make the Work available on an Open Access basis (and intends to pay the relevant APC), the terms of reuse of such Open Access shall be governed by a Creative Commons licence – details of these licences and which Creative Commons licence will apply to this Work are set out in our licence referred to above.

Other than as permitted in any relevant BMJ Author's Self Archiving Policies, I confirm this Work has not been accepted for publication elsewhere, is not being considered for publication elsewhere and does not duplicate material already published. I confirm all authors consent to publication of this Work and authorise the granting of this licence.

For peer review only

TITLE PAGE

Title

Total and direct associations between high temperatures and preterm births in Detroit, Michigan

Authors

Carina J. Gronlund[1], Alyssa J. Yang[2], Kathryn C. Conlon[3], Rachel S. Bergmans[4], Hien Q. Le[5], Stuart A. Batterman[6], Robert L. Wahl[7], Lorraine Cameron[7], Marie S. O'Neill[6,8]

[1]Corresponding author:
University of Michigan Institute for Social Research
Survey Research Center, Social Environment and Health Program
426 Thompson St. Rm 3340
Ann Arbor, MI 48104
gronlund@umich.edu
734-615-9215

[2]Urban Indian Health Institute, Seattle, WA, USA

[3]University of California at Davis, Davis, CA, USA

[4]Department of Psychiatry, University of Michigan, Ann Arbor, MI, USA

[5]Health Services/Epidemiology, The Chemours Company, Wilmington, DE, USA

[6]Department of Environmental Health Sciences, University of Michigan, Ann Arbor, MI, USA

[7]Michigan Department of Health and Human Services, Lansing, MI, USA

[8]Department of Epidemiology, University of Michigan, Ann Arbor, MI, USA

Word count: 3,815

For peer review only - http://bmjopen.bmj.com/site/about/guidelines.xhtml

ABSTRACT

Background: Preterm births (PTBs) represent significant health risks, and several studies have found associations between high outdoor temperatures and PTB. We estimated both the total and natural direct effects (independent of particulate matter, ozone and nitrogen dioxide air pollutants) of the prior two-day mean apparent temperature (AT) on PTB in the Detroit, Michigan area for the warm months (May to September), 1991-2001. We evaluated effect modification by maternal age, race, education, smoking status, and prenatal care.

Methods: We used a time series Poisson regression with splines of AT, wind speed, solar radiation, and citywide average precipitation to estimate total effects. To accommodate multiple mediators and exposure-mediator interactions, AT inverse odds weights, predicted by meteorological and air pollutant covariates, were added in a subsequent model to estimate direct effects.

Results: At 24.9 °C relative to 18.6 °C, 10.6% (95% Confidence Interval: 3.8%, 17.2%) of PTBs were attributable to the total effects of AT, and 10.4% (95% CI: 2.2%, 17.5%) to direct effects. Relative excess risks of interaction indicated that the risk of PTB with increasing temperature above 18.6 °C was significantly lower among black mothers and higher among mothers less than 19, older than 30, with late or no prenatal care, and who smoked.

Conclusion: This additional evidence of a direct association between high temperature and PTB may motivate public health interventions to reduce extreme heat exposures among pregnant women, particularly among those who may have enhanced vulnerability.

For peer review only - http://bmjopen.bmj.com/site/about/guidelines.xhtml

For peer review only

STRENGTHS AND LIMITATIONS OF THIS STUDY

- We expand on a growing literature demonstrating total effects of temperature on PTB by examining mediation of the total temperature effect by air pollutants.
- We did not have geographic identifiers for the study participants at a level as fine as residential address or indoor exposure information, and we therefore assigned time-varying environmental exposures at an outdoor, citywide level. However, a low prevalence of residential air conditioning (32%) in the study time period suggests that outdoor exposures may have more accurately reflected indoor exposures than in more recent studies.
- We account for multiple mediators and potential exposure-mediator interactions by applying a straightforward technique—the use of inverse odds weights—to separate the total effects of temperature on PTB from the direct effects.
- We used methods examining potential synergistic effects, as opposed to just examining statistical interactions, by other characteristics that confer vulnerability to PTB.

For peer review only

For peer review only - http://bmjopen.bmj.com/site/about/guidelines.xhtml

INTRODUCTION

Preterm births (PTBs) are defined as births that occur before 37 gestational weeks. Babies born prematurely are at greater risk of infant death and impaired health, such as cerebral palsy and impairments of cognitive development, hearing, vision, respiration, and digestion. In 2016, 10% of babies were born prematurely, with statewide rates ranging 7.8-13.7%[1]. Within-state disparities in preterm birth rates can be greater: from 1990-2010, 18% of births in Detroit, Michigan were PTBs[2].

Certain risk factors are known to be associated with PTB, such as cigarette smoking, alcohol use, hypertension, and diabetes[1]. Air pollution is associated with PTB[3 4], and higher ambient temperature has been proposed as a risk factor for PTB[5 6].

High ambient temperature could pose a risk for PTB through several biological pathways, including stress and dehydration pathways.[789] Several studies have evaluated possible associations between high ambient temperature and PTB, with mixed results[5 6]. Studies in cities in Australia, Quebec, China, Belgium, Italy, Spain and across the U.S. have all found significant positive associations between hot temperatures within the week preceding delivery and PTB[5 6 10-18]. High ambient temperatures in the month of conception and the third trimester were also positively associated with PTB in Changsha, China {Zheng, 2018 #3222;Zhong, 2018 #3223}. However, earlier, rigorously conducted time series analyses of temperature and PTB in Germany and in London, England did not find associations[19 20]. Likewise, in Chicago, Illinois, Porter et al. found no effect of the July 1995 heat wave on gestational length[21]. Sources of heterogeneity among these effect estimates may include differences in study design, prevailing climate and regional adaptation, population structure, exposure assessment, critical windows of exposure considered, and methods of gestational age assessment[14].

For peer review only - http://bmjopen.bmj.com/site/about/guidelines.xhtml

Studies often control for air pollution exposure, although the argument has been made that treating air pollutants as confounders of temperature-health associations is inappropriate given that high temperatures can contribute to increased concentrations of some air pollutants, and both temperature and air pollution are affected by sunlight[22]. Differences in the manner in which air pollutants are accounted for in the temperature-PTB modeling may also account for some of the heterogeneity observed in this literature.

We investigated the association between high AT and PTB in the Detroit, Michigan area, estimating both the total effects of temperature as well as the natural direct effects, excluding potential mediation effects by the air pollutants ozone, particulate matter with an aerodynamic diameter of less than 10 micrometers (PM_{10}), and nitrogen dioxide (NO_2). We further examined whether the maternal risk factors of age, race, education, smoking, and level of prenatal care modified the association between high AT and PTB.

METHODS

Outcome Variable

Birth outcome data were derived from birth certificate records kept by the Michigan Department of Health and Human Services[4]. This sample population included live, singleton births that occurred from January 1, 1991 to December 31, 2001 in ZIP codes within 4 km of one of three air quality monitors (Allen Park, Linwood, and East 7 Mile) in and bordering Detroit, Michigan[4]. In the final data set, births were categorized as term vs. PTB, defined as births that were less than 37 gestational weeks. We limited the data set to births that occurred May-September, in order to focus on heat exposures.

Identification of Confounders and Mediators

A directed acyclic graph (DAG) was constructed to define the causal framework and elucidate potential mediating effects of air pollutants in the association between AT and PTB

For peer review only - http://bmjopen.bmj.com/site/about/guidelines.xhtml

(Figure 1). In this DAG, AT is the exposure of interest and PTB the outcome of interest. Given suspected associations between PTB and precipitation [23] and between PTB and vitamin D, for which one source is solar radiation [24], as well as causal meteorological relationships between AT, solar radiation, and precipitation via a parent weather system, precipitation and solar radiation are potential confounders of the AT-PTB association. In this DAG, primary and secondary air pollutants are the hypothesized mediators of the temperature-PTB association given that: 1) they have been associated with the health outcome in other locations[3] and in this dataset in the third trimester[4] , 2) high temperature enhances the rate of ground-level ozone formation, and 3) power plant emissions increase when temperatures rise to accommodate additional demand for air conditioning[25]. Furthermore, the weather system is a causal parent to both AT and the pollutants and therefore a potential confounder given that meteorological conditions such as wind speed, precipitation, and cloud cover can affect pollutant formation and exposure as well as sunlight, which increases AT and promotes ozone formation[26]. Therefore, a model characterizing the *total effect* of AT would need to include the confounders of solar radiation, wind speed, and precipitation but not include pollutants. A model characterizing the *direct effect* of AT, independent of air pollution mediation, would need to include these mediators. The modeling used to characterize direct and indirect effects is further described in the Statistical Analysis section below.

Exposure Variables

Daily mean temperature, dew point temperature, and wind speed were obtained from the National Center for Environmental Information[27]. Data from the Detroit City Airport was used due to its proximity to the mothers' ZIP codes of residence. To better represent thermal discomfort, we used apparent temperature (AT) rather than air temperature. AT is similar to heat index, and increases with both air temperature and relative humidity. AT was calculated using

For peer review only - http://bmjopen.bmj.com/site/about/guidelines.xhtml

the following formula: [-2.653 + (0.994*Temperature in °C) + 0.0153*(Dew Point Temperature in °C)^2][28].[29] From Detroit City Airport, 11% and 15% of temperatures and dew points were missing, respectively, so data from Detroit Metropolitan Airport were used to replace these missing data. Among non-missing values, daily AT from Detroit City Airport was highly correlated with that from Detroit Metropolitan Airport (Pearson's correlation coefficient = 0.98).

The total amount of direct and diffuse solar radiation received on a horizontal surface during each 60-minute period at the Detroit City Airport was retrieved from the National Solar Radiation Database[30]. These data were modeled from meteorological data including cloud cover, aerosol, and ozone data from sources such as sun photometers, satellites and albedo data[31]. We further estimated daily means from the hourly solar radiation values.

Daily precipitation totals were obtained from Oregon State University's PRISM Climate Group[32]. These are modeled at a 4-km resolution based on observations and a climatologically-aided interpolation process. Rasters were cropped to the City of Detroit and daily citywide averages were calculated.

Daily eight-hour maximum ozone and daily mean NO_2 and PM_{10} concentrations were obtained from the Environmental Protection Agency for all Wayne County, Michigan monitors and averaged by day and pollutant[33]. Only daily monitor values for which at least a single daily or 18 of the 24-hourly values were available were retained, resulting in substantial missingness, which was addressed in the statistical analysis below.

Birth certificate data also included date of birth and maternal age group (16-19, 20-29, and over 30), race (black or white), smoking status (smoker vs. non-smoker), education level (less than high school vs. high school or higher), and level of prenatal care (prenatal care vs. late or no prenatal care), which were used in analyses of effect modification.

For peer review only - http://bmjopen.bmj.com/site/about/guidelines.xhtml

Patient and Public Involvement

Patients were not involved. This research was deemed exempt from review by the University of Michigan Institutional Review Board.

Statistical Analysis

Case-crossover analysis is commonly used in studies of PTB and temperature[14 15 34]. Because PTB is not a rare event in this particular population, the case-crossover odds ratios would not approximate risk ratios. Therefore, we used a time series design with Poisson regression, controlling for seasonal and long-term variations in PTB counts with a cubic b-spline for day-of-year, with 5, 2 or 8 knots, and a cubic b-spline for year with 2 knots. AT exposure was characterized as two-day mean AT, or the mean of the AT values on the day of and the day before birth. For AT and the covariates, non-linearity was considered by initially modeling each as a b-spline with three knots and selecting a single knot for subsequent modeling of the covariate as a piecewise linear spline when substantial nonlinearity was visually evident. The 95% confidence intervals (CIs) were constructed from the 2.5^{th} and 97.5^{th} percentiles of 500 bootstrapped samples. In sensitivity analyses, given its wide usage for this research question, a time-stratified case-crossover design was used with time-strata defined as two- or three-week periods.

To account for missing air pollutant values, we conducted multiple imputations using chained equations. We used a more general model including lag days 0-2 of the above meteorological and pollutant values. The air pollutant values were well-predicted by lag days 0-2 meteorology and air pollution values, and from examinations of trace plots, or scatter plots of successive parameter estimates, we determined that a total of three imputations following two burn-ins was sufficient. All subsequent analyses were conducted on each of the three resulting data sets.

For peer review only - http://bmjopen.bmj.com/site/about/guidelines.xhtml

To estimate the total effects of AT, we included terms for solar radiation, wind speed, and precipitation in the Poisson model, which blocked all of the paths in the DAG from AT to PTB that did not pass through the mediators (Figure 1). However, to estimate natural direct effects, we used a more generalizable technique—inverse odds weighting—given that we had multiple mediators and as well as potential exposure-mediator interactions[35]. In this technique, we first fit a standard linear regression of two-day mean AT (meanAT01) on the air pollutant mediators and covariates (solar radiation, wind speed, precipitation). We then estimated:

inverse odds = 1 / exp(predicted meanAT01 / σ^2)   Equation 1

where σ^2 was the model mean squared-error. The inverse odds were then used as weights in the subsequent analysis of PTB and AT with the other covariates but not the air pollutants. The weights render AT independent of the mediators, thereby allowing the estimation of AT separately from the effects of the air pollutant mediators on PTB. Total and direct effects were calculated from each of the three imputed data sets and then each averaged.

To assess the public health impact of AT on PTB, we estimated the attributable fraction (AF), or the percent of PTB attributable to high AT among women exposed to the high AT. The AF was estimated as: 1 / (1 – RR) * 100% where the RR was for the 95th percentile (24.9 °C) relative to the 50th percentile (18.6 °C) of May-September two-day mean AT. Indirect effects were estimated as the difference between the total effects and the direct effects. The 95% confidence intervals (CIs) were constructed from the 2.5th and 97.5th percentiles of 500 bootstrapped samples.

To understand how the effects varied among maternal subgroups, or effect modification of the PTB-AT association, we simultaneously included interaction terms between the AT spline and the indicator variables for black race, age 16-19, age 30 years or older, low prenatal care

For peer review only - http://bmjopen.bmj.com/site/about/guidelines.xhtml

(late or no prenatal care), current smoker, and no high school education. We expanded the data set such that we had rows for each unique combination of date and daily exposures, race, age group, education, prenatal care, and smoking status. Given the large number of zero counts, we specified a negative binomial distribution rather than a Poisson distribution. For public health significance, we were interested in the absolute rather than the relative increase in PTB risk due to synergistic effects between temperature and each modifier of interest. Therefore we focused on a*additive*, rather than the*multiplicative*, interactions. Relative excess risk due to interaction (RERI)[36] was calculated for each potential modifier as:

$$RR_{AT=95th,EM=1} - RR_{AT=95th,EM=0} - RR_{AT=50th,EM=1} + 1 \qquad \text{Equation 2}$$

where EM was the effect modifier of interest and the denominator of each RR was the risk at the 50th percentile of AT (18.6 °C) and the absence of the modifier (EM = 0). RERI confidence CIs were bootstrapped from 1,000 samples. Analyses were conducted in SAS 9.4 (Copyright 2016, SAS Institute Inc., Cary, NC, USA) using PROC GLIMMIX, which allows for multiple splines in a Poisson regression. Figure 2 was constructed in R (R Foundation for Statistical Computing, Vienna, Austria) using the dlnm package[37] following modeling using the glm.nb function in the MASS package[38].

RESULTS

There were 9,053 singleton PTBs in this Detroit-area sample, May-September, 1991-2001 (Table 1). There were fewer PTBs in September compared to the other months, and consistent with Detroit's population decline, the number of PTBs declined with time. Considering individual characteristics, 27.0% of the PTBs were among white mothers and 71.1% were among black mothers. A majority of the mothers were non-smokers (73.1%), had prenatal care (55.6%), were 20-29 years of age (53.2%) and had at least a high school education (60.2%) (Table 1).

For peer review only - http://bmjopen.bmj.com/site/about/guidelines.xhtml

On average, AT exposure was 18.0 °C, and among the days when AT was greater than the time-period-specific daily median of 18.6 °C (i.e., for the upper end of the temperature spline), AT averaged 1.5 °C higher. A total of 53% of the cases occurred on days with no rain, so the geometric mean precipitation was 0.4 mm. The mean ozone concentration was 44.8 ppb and the maximum was 102 ppb (Table 2). In examining the daily time series of ozone over the study period, the three-year running averages of the fourth highest daily 8-hour maximum value of ozone ranged from 77 to 92 ppb, suggesting that the (current) National Ambient Air Quality Standards (70 ppb) had been exceeded in each year of the study.

In a crude model of the association between PTB and AT where AT was modeled flexibly as a b-spline with 3 df, we found a nonlinear association, with approximately null effects below 18-19 C and an increasingly stronger positive association at higher temperatures (Figure 2). To address potential sensitivity of the results to control for season, we varied the df in the day-of-year term, using 2, 5, and 8 df. The results were not highly sensitive to this choice, with the point estimates of the percentages of PTB attributable to AT ranging only from 8.1% (8 df) to 9.9% (2 df) among pregnant women exposed to days when AT was 24.9 °C vs exposure on 18.6 °C days (Table 3). In case-crossover analyses, the resulting odds ratios were also statistically significantly greater than 1.0, regardless of whether the time strata were two-week, three-week, or one-month periods (Supplemental Material Table S1). However, the odds ratios were greater in magnitude than the risk ratios estimated in the time series design, which is expected, given that odds ratios overestimate risk ratios when events are not rare.

The total effects were estimated as 10.6% (95% CI: 3.8%, 17.2%) of PTBs attributable to AT among pregnant women exposed to a day when AT was 24.9 °C vs. exposure on an 18.6 °C day. Accounting for mediation of this effect by PM_{10}, ozone, and NO_2 by using inverse odds

For peer review only - http://bmjopen.bmj.com/site/about/guidelines.xhtml

weights, the direct effect of AT on preterm birth was decreased to 10.4% (95% CI: 2.2%, 17.5%), although the difference between the two values was not statistically significant (indirect effect = 0.3%, 95% CI: -1.7%, 2.6%).

In examining the RERI from interactions between AT and maternal characteristics, black race was found to be protective, with an RERI significantly less than 0 (Table 4). The magnitudes of RERIs are not meaningful, but this result indicates that the association between high temperature and PTB is weaker among women of black race than non-black race. In contrast, RERIs for age 16-19 years, age > 30 years, low prenatal care and tobacco smoking were all significantly greater than 0, indicating that the association between PTB and high temperature is stronger among women with these characteristics.

DISCUSSION

The strong association between short-term temperature exposure and PTB in Detroit, Michigan was consistent with several other studies' observed associations between PTB or diminished gestational age and temperature exposures within the week prior to delivery at regionally high temperatures. Regions in which these associations have been found include Central Australia (8.3% at 40 °C daily maximum temperature)[13], Northern California, USA (11.6% increase for a 5.6 °C increase in weekly average AT in the warm season)[34], Alabama, USA (32.4% increase with two consecutive days of daily mean temperatures above the 98th percentile)[39], Barcelona, Spain (5-day reduction in average gestational age with heat index above the 99th percentile)[10], Rome, Italy (1.9% increase per 1 °C increase in maximum AT in the prior two days and 19% increase during heat waves)[40], Brisbane, Australia (13%-100% increase during heat waves), in an aggregated sample of 12 U.S. cities (12-16% increase with 2.8 °C increase in prior week)[14], in Southern China (7% increase with previous-week temperatures

For peer review only - http://bmjopen.bmj.com/site/about/guidelines.xhtml

above 95th percentile)[12], and in a multi-city USA sample (2% increased PTB risk with extreme heat in the prior 4 days) [41].

The precise biological mechanisms by which high ambient temperature might increase risk for PTB are unclear, although psychosocial stress[7] and dehydration pathways are plausible.[7]. Stress increases the levels of cortisol and epinephrine, potentially leading to the secretion of placental corticotropin-releasing hormones (CRH). Placental CRH can then activate the fetal hypothalamic-pituitary-adrenal (HPA) axis, which could prompt the fetal expression of cortisol and dehydroepiandrosterone-sulfate (DHEA-S) and placental release of estriol and prostaglandins, potentially triggering PTB[8]. Dehydration due to high temperatures and sweating could also reduce blood flow to the uterus and induce a greater release of antidiuretic hormone and oxytocin, which could trigger labor onset[9].

One strength of our study may be a tighter correspondence between the true ambient temperatures experienced by our sample and the measured outdoor ambient AT given the low air conditioning prevalence in the Detroit area during the time period. Specifically, in 1993, the last year for which county-specific American Housing Survey data were available in the Detroit area, only 32% of Wayne County households had central air conditioning[42]. Furthermore, central air conditioning prevalence was low in this region despite a “hot-summer humid continental climate” Köppen Climate Classification[43], allowing a fairly wide range of warm season AT exposure over which AT-PTB associations could be examined. In contrast, regions where associations between temperature and PTB were null in rigorously conducted daily time series studies included Brandenburg and Saxony, Germany[20] and London, UK[19], which are in “temperate oceanic climates,” where all months have average temperatures below 22 °C [43]. Additionally, Guo et al.[12] found an association between previous-week temperature and PTB

For peer review only - http://bmjopen.bmj.com/site/about/guidelines.xhtml

only in the "hot" region of China, defined as having annual average temperatures 19 °C or higher. Furthermore, in a survival analysis of previous-week temperature and PTB in 18 European cities, the pooled effect estimates were null, and no individual city results were presented[44]. Again, with the exception of 4 cities, these European cities were all in cooler climates where all months have average temperatures below 22 °C[43]. This suggests that in regions where the threat of extremely high temperatures is rare, PTB is not triggered at warm temperatures, regardless of relative temperature thresholds, even at the same absolute temperatures at which heat-associated PTB is experienced in warmer climates. This could be due to differences in emotional stress[45] or physiological stress responses to warm temperatures between climates. Alternatively, the heterogeneity in effect estimates could be due to regional differences in the misclassification of the true individually-experienced temperatures by outdoor temperatures. Finally, in the 18-city European study, the PTB rate was only 5%, suggesting that PTB etiologies may differ between Europe and the U.S., in which case the pregnancies in the European cohorts could have been less susceptible physiologically to high temperature[44].Other strengths of our study include our consideration of near-surface solar radiation and weather conditions as confounders of the total effects of AT, air pollutants as mediators rather than confounders, and estimation of the direct effects of AT as distinct from those mediated by air pollutants. By using an analysis technique that was "agnostic" to exposure-mediator interactions and could accommodate multiple mediators[35], we found that most if not all of the effect of AT on PTB is direct, and not mediated by daily changes in air pollution concentrations. However, this mediation analysis technique tends to over-estimate the confidence intervals around the indirect effects[35], which may account for our finding a null indirect effect of air pollutants on PTB when previous research has in fact identified significant associations between short-term increases in

For peer review only - http://bmjopen.bmj.com/site/about/guidelines.xhtml

air pollution and PTB[46]. We may have also underestimated the air pollutant effects because we were only examining the indirect effects of AT through an air pollutant pathway rather than the total effects of air pollution and because we only considered PM_{10} rather than $PM_{2.5}$.

Another strength of our study is that we reported RERIs, rather than merely reporting statistical interactions in the models. In doing so, we provided evidence for synergistic effects of high temperature with the independent risk factors for PTB of prenatal care, smoking status, and age. Interestingly, in this majority-black population, the risk of PTB with high temperatures was actually lower among black mothers, after controlling for age, education, smoking status, and prenatal care.

Limitations include the fact that we were not able to distinguish between spontaneous and medically indicated PTBs. Considering that medically indicated preterm deliveries are unlikely to be related to temperature, this limitation affects the generalizability of our relative risks to populations where the relative percentages of spontaneous vs. medically indicated PTB differ. We also did not have information on how much earlier the birth was, e.g., a one-day premature birth vs. a six-week premature birth. This prevented us from including an offset for the population of pregnancies at-risk of PTB[47]. Our model included a spline for year and spline for day-of-year to attempt to capture within-season variations in PTBs. Varying the degrees of freedom in the day-of-year term from 2 to 8 had only a mild effect on the relative risks, although this would not have captured sub-weekly changes in PTBs. Another limitation is our dependence on last menstrual period rather than ultrasound-derived gestational age. Last menstrual period tends to result in more births being classified as preterm, particularly among Blacks[48]. This limitation, assuming it was not correlated with temperature, would bias our results towards the null.

For peer review only - http://bmjopen.bmj.com/site/about/guidelines.xhtml

An additional limitation is that the prevalence of characteristics enhancing vulnerability to PTB may have changed since the study period, thereby decreasing the generalizability of these results to the present-day population of this region. Future research should employ present-day cohorts of pregnant women, for which a denominator of total pregnancies is therefore available, linked with refined temperature exposure measurements, including indoor and neighborhood temperature exposure estimates. These research refinements will better characterize the thermal exposures and severity of heat-induced PTB, or more specifically, gestational length, and better identify which pregnancies are particularly vulnerable to early parturition on hot days.

Despite the aforementioned limitations, given the evidence from this and other studies, pregnant women, in addition to older adults, should be considered as a group vulnerable to short-term heat health effects when considering housing and climate adaptation measures in Detroit and similar or warmer climates.

For peer review only

For peer review only - http://bmjopen.bmj.com/site/about/guidelines.xhtml

CONTRIBUTORSHIP STATEMENT

MSO, SAB, HQL, RLW, and LC obtained the data or resources, critiqued the analysis, and reviewed the manuscript. RSB and KCC assisted with preliminary analyses, critiqued the analysis and reviewed the manuscript. AJY performed and drafted preliminary analyses and reviewed the manuscript. CJG directed preliminary analyses, revised the analysis, and revised the manuscript. We thank Leah Comment for early-stage data management and analysis assistance. We also thank Patricia Maina for her contribution to the preterm birth and heat literature review and Sung Kyun Park for early stage advice.

FUNDING

This work was supported by a Michigan Institute for Clinical and Health Research Postdoctoral Translational Scholars Fellowship (2UL1TR000433-06), National Institute of Environmental Health Sciences grants K99ES026198 and P30ES017885, Cooperative Agreement Number EH001124 from the Centers for Disease Control and Prevention (CDC), National Science Foundation grant 1520803, and CDC/ National Institute for Occupational Safety and Health grant T42 OH008455. Its contents are solely the responsibility of the authors and do not necessarily represent the official views of the CDC or the Michigan Department of Health and Human Services. None of the funders participated in the design, collection, analysis, or interpretation of the data.

COMPETING INTERESTS

None declared.

DATA SHARING STATEMENT

Birth outcome data were derived from birth certificate records kept by the Michigan Department of Health and Human Services (MDHHS).[4] These confidential data may be obtained from the MDHHS Division for Vital Records and Health Statistics following completion of a data use agreement and IRB approval. The exposure data are publicly available and were obtained as described in the Methods. The processed exposure data may be requested from C. Gronlund (gronlund@umich.edu).

For peer review only

For peer review only - http://bmjopen.bmj.com/site/about/guidelines.xhtml

REFERENCES

1. Centers for Disease Control and Prevention (CDC). Premature Birth 2017 [updated 11/6/2017. Available from: https://www.cdc.gov/features/prematurebirth/ accessed Jan 29 2018.
2. Make Your Date Detroit, Center for Advanced Obstetrical Care and Research, Wayne State University. Why This Matters--Facts 2017 [Available from: https://makeyourdate.org/facts/ accessed Jan 29 2018.
3. Sun XL, Luo XP, Zhao CM, et al. The association between fine particulate matter exposure during pregnancy and preterm birth: a meta-analysis. *BMC Pregnancy Childbirth* 2015;15:12. doi: 10.1186/s12884-015-0738-2
4. Le HQ, Batterman SA, Wirth JJ, et al. Air pollutant exposure and preterm and term small-for-gestational-age births in Detroit, Michigan: Long-term trends and associations. *Environment International* 2012;44:7-17. doi: http://dx.doi.org/10.1016/j.envint.2012.01.003
5. Carolan-Olah M, Frankowska D. High environmental temperature and preterm birth: a review of the evidence. *Midwifery* 2014;30(1):50-9. doi: 10.1016/j.midw.2013.01.011 [published Online First: 2013/03/12]
6. Strand LB, Barnett AG, Tong S. The influence of season and ambient temperature on birth outcomes: a review of the epidemiological literature. *Environ Res* 2011;111(3):451-62. doi: 10.1016/j.envres.2011.01.023 [published Online First: 2011/02/22]
7. McMorris T, Swain J, Smith M, et al. Heat stress, plasma concentrations of adrenaline, noradrenaline, 5-hydroxytryptamine and cortisol, mood state and cognitive performance. *International Journal of Psychophysiology* 2006;61(2):204-15. doi: https://doi.org/10.1016/j.ijpsycho.2005.10.002
8. Behrman RE, Adashi EY, Allen MC, et al. Preterm Birth: Causes, Consequences, and Prevention. In: Medicine Io, ed. Report Brief. Washington, DC: Institute of Medicine, 2006.
9. Stan C, Boulvain M, Hirsbrunner-Amagbaly P, et al. Hydration for treatment of preterm labour. *Cochrane Database Syst Rev* 2002;2
10. Dadvand P, Basagana X, Sartini C, et al. Climate extremes and the length of gestation. *Environ Health Perspect* 2011;119(10):1449-53. doi: 10.1289/ehp.1003241 [published Online First: 2011/06/11]
11. Strand LB, Barnett AG, Tong S. Maternal exposure to ambient temperature and the risks of preterm birth and stillbirth in Brisbane, Australia. *Am J Epidemiol* 2012;175(2):99-107. doi: 10.1093/aje/kwr404 [published Online First: 2011/12/15]
12. Guo TJ, Wang YY, Zhang HG, et al. The association between ambient temperature and the risk of preterm birth in China. *Sci Total Environ* 2018;613:439-46. doi: 10.1016/j.scitotenv.2017.09.104
13. Mathew S, Mathur D, Chang AB, et al. Examining the Effects of Ambient Temperature on Pre-Term Birth in Central Australia. *International Journal of Environmental Research and Public Health* 2017;14(2) doi: 10.3390/ijerph14020147
14. Ha SD, Liu DP, Zhu YY, et al. Ambient Temperature and Early Delivery of Singleton Pregnancies. *Environmental Health Perspectives* 2017;125(3):453-59. doi: 10.1289/ehp97

For peer review only - http://bmjopen.bmj.com/site/about/guidelines.xhtml

15. Basu R, Chen H, Li DK, et al. The impact of maternal factors on the association between temperature and preterm delivery. *Environmental Research* 2017;154:109-14. doi: 10.1016/j.envres.2016.12.017
16. Schifano P, Asta F, Dadvand P, et al. Heat and air pollution exposure as triggers of delivery: A survival analysis of population-based pregnancy cohorts in Rome and Barcelona. *Environment International* 2016;88:153-59. doi: 10.1016/j.envint.2015.12.013
17. He JR, Liu Y, Xia XY, et al. Ambient Temperature and the Risk of Preterm Birth in Guangzhou, China (2001-2011). *Environ Health Perspect* 2015 doi: 10.1289/ehp.1509778 [published Online First: 2015/12/17]
18. Arroyo V, Diaz J, Ortiz C, et al. Short term effect of air pollution, noise and heat waves on preterm births in Madrid (Spain). *Environmental Research* 2016;145:162-68. doi: 10.1016/j.envres.2015.11.034
19. Lee SJ, Hajat S, Steer PJ, et al. A time-series analysis of any short-term effects of meteorological and air pollution factors on preterm births in London, UK. *Environ Res* 2008;106(2):185-94. doi: 10.1016/j.envres.2007.10.003 [published Online First: 2007/11/21]
20. Wolf J, Armstrong B. The association of season and temperature with adverse pregnancy outcome in two German states, a time-series analysis. *PLoS One* 2012;7(7):e40228. doi: 10.1371/journal.pone.0040228 [published Online First: 2012/07/14]
21. Porter KR, Thomas SD, Whitman S. The relation of gestation length to short-term heat stress. *American Journal of Public Health* 1999;89(7):1090-92. doi: 10.2105/ajph.89.7.1090
22. Buckley JP, Samet JM, Richardson DB. Commentary: Does air pollution confound studies of temperature? *Epidemiology* 2014;25(2):242-5. doi: 10.1097/ede.0000000000000051 [published Online First: 2014/02/04]
23. Yu X, Feric Z, Cordero JF, et al. Potential influence of temperature and precipitation on preterm birth rate in Puerto Rico. *Scientific reports* 2018;8:9. doi: 10.1038/s41598-018-34179-z
24. Dovnik A, Mujezinović F. The Association of Vitamin D Levels with Common Pregnancy Complications. *Nutrients* 2018;10(7):867. doi: 10.3390/nu10070867
25. Abel D, Holloway T, Kladar RM, et al. Response of Power Plant Emissions to Ambient Temperature in the Eastern United States. *Environ Sci Technol* 2017;51(10):5838-46. doi: 10.1021/acs.est.6b06201
26. Environmental Protection Agency (EPA). Ozone Pollution and Your Patient's Health: What is Ozone? 2018 [Available from: https://www.epa.gov/ozone-pollution-and-your-patients-health/what-ozone accessed December 20 2018.
27. National Climatic Data Center. Integrated Surface Database Lite 2010 [Available from: http://www.ncdc.noaa.gov/oa/climate/isd/index.php accessed July 2010.
28. Kalkstein LS, Valimont, K.M. An evaluation of summer discomfort in the United States using a relative climatological index. *Bull Am Meteorol Soc* 1986;7:842-48.
29. Oswald EM, Rood RB, Zhang K, et al. An investigation into the spatial variability of near-surface air temperatures in the Detroit, MI metropolitan region. *J Appl Meteorol Climatol* 2012;51(7):1290-304. doi: 10.1175/JAMC-D-11-0127.1
30. National Solar Radiation Database, 1991-2010 Update [Available from: https://rredc.nrel.gov/solar/old_data/nsrdb/ accessed August 25 2017.

For peer review only - http://bmjopen.bmj.com/site/about/guidelines.xhtml

31. Wilcox S. National Solar Radiation Database 1991-2010 Update: User's Manual. In: National Renewable Energy Laboratory, ed.: U.S. Department of Energy, Office of Energy Efficiency & Renewable Energy, 2012.
32. PRISM Climate Group. PRISM Climate Data 2016 [Available from: http://www.prism.oregonstate.edu/ accessed March 6 2016.
33. U.S. Environmental Protection Agency. AirData 2015 [Available from: http://aqsdr1.epa.gov/aqsweb/aqstmp/airdata/download_files.html accessed Feb 5 2015.
34. Avalos LA, Chen H, Li DK, et al. The impact of high apparent temperature on spontaneous preterm delivery: a case-crossover study. *Environmental Health* 2017;16 doi: 10.1186/s12940-017-0209-5
35. Nguyen QC, Osypuk TL, Schmidt NM, et al. Practical Guidance for Conducting Mediation Analysis With Multiple Mediators Using Inverse Odds Ratio Weighting. *American Journal of Epidemiology* 2015;181(5):349-56. doi: 10.1093/aje/kwu278
36. Nie L, Chu H, Li F, et al. Relative Excess Risk Due to Interaction: Resampling-based Confidence Intervals. *Epidemiology* 2010;21(4):552-56.
37. Gasparrini A, Armstrong B, Kenward MG. Distributed lag non-linear models. *Stat Med* 2010;29(21):2224-34. doi: 10.1002/sim.3940 [doi] [published Online First: 2010/09/03]
38. Venables WN, Ripley BD. Functions and Datasets to Support "Modern Applied Statistics with S" (4th edition, 2002) 2018 [Available from: https://cran.r-project.org/web/packages/MASS/index.html accessed April 1 2019.
39. Kent ST, McClure LA, Zaitchik BF, et al. Heat Waves and Health Outcomes in Alabama (USA): The Importance of Heat Wave Definition. *Environ Health Perspect* 2013 doi: 10.1289/ehp.1307262 [published Online First: 2013/11/26]
40. Schifano P, Lallo A, Asta F, et al. Effect of ambient temperature and air pollutants on the risk of preterm birth, Rome 2001-2010. *Environ Int* 2013;61:77-87. doi: 10.1016/j.envint.2013.09.005 [published Online First: 2013/10/10]
41. Sun SZ, Weinberger KR, Spangler KR, et al. Ambient temperature and preterm birth: A retrospective study of 32 million US singleton births. *Environment International* 2019;126:7-13. doi: 10.1016/j.envint.2019.02.023
42. United States. Bureau of the Census. American Housing Survey, 1993: MSA Core and Supplement File: [distributor], 2006.
43. Peel MC, Finlayson BL, McMahon TA. Updated world map of the Köppen-Geiger climate classification. *Hydrol Earth Syst Sci* 2007;11(5):1633-44. doi: 10.5194/hess-11-1633-2007
44. Giorgis-Allemand L, Pedersen M, Bernard C, et al. The Influence of Meteorological Factors and Atmospheric Pollutants on the Risk of Preterm Birth. *American Journal of Epidemiology* 2017;185(4):247-58. doi: 10.1093/aje/kww141
45. Lin YF, Hu WJ, Xu J, et al. Association between temperature and maternal stress during pregnancy. *Environmental Research* 2017;158:421-30. doi: 10.1016/j.envres.2017.06.034
46. Stieb DM, Lavigne E, Chen L, et al. Air pollution in the week prior to delivery and preterm birth in 24 Canadian cities: a time to event analysis. *Environ Health* 2019;18(1):1. doi: 10.1186/s12940-018-0440-8 [published Online First: 2019/01/05]
47. Vicedo-Cabrera AM, Iniguez C, Barona C, et al. Exposure to elevated temperatures and risk of preterm birth in Valencia, Spain. *Environmental Research* 2014;134:210-17. doi: 10.1016/j.envres.2014.07.021

For peer review only - http://bmjopen.bmj.com/site/about/guidelines.xhtml

48. Wingate MS, Alexander GR, Buekens P, et al. Comparison of Gestational Age Classifications: Date of Last Menstrual Period vs. Clinical Estimate. *Annals of epidemiology* 2007;17(6):425-30. doi: https://doi.org/10.1016/j.annepidem.2007.01.035

For peer review only

For peer review only - http://bmjopen.bmj.com/site/about/guidelines.xhtml

TABLES

Table 1. Demographics of Preterm Births in Detroit, MI Study Area, May-September, 1991-2001.

	N	%
Total	9,053	100
Month		
May	1,918	21.2
June	1,944	21.5
July	1,903	21.0
August	1.754	19.4
September	1,534	16.9
Year (Two Equal Time Periods)		
1991-1995	4,708	56.2
1996-2000	3,670	43.8
Maternal Race		
White	2,443	27.0
Black	6,433	71.1
Other	177	1.9
Maternal Smoking Status		
Non-Smoker	6,621	73.1
Smoker	2,345	25.9
Missing	87	1.0
Level of Prenatal Care		
Prenatal Care	5,037	55.6
Late or No Prenatal Care	2,960	32.7
Missing	1,056	11.7
Maternal Age		
16-19 years	1,782	19.7
20-29 years	4,821	53.2
≥ 30 years	2,450	27.1
Maternal Education		
Less than High School	3,485	38.5
High School or Higher	5,451	60.2
Missing	117	1.3

For peer review only - http://bmjopen.bmj.com/site/about/guidelines.xhtml

Table 2. Daily Exposures Among the PTB Cases, Detroit, MI Area, May-September, 1991-2001.

	N	Median	Mean	Min	Max
Two-day mean apparent temperature, °C	9,053	18.6	18.0	3.1	28.0
Two-day mean apparent temperature 18.6 °C and above	9,053	0.1	1.5	0.0	9.4
Mean solar radiation (W/m^2)	9,053	226.4	217.7	38.1	352.8
Total precipitation (mm)[a]	9,053	0.0	0.4	0.0	56.7
Mean wind speed (m/s)	9,053	3.6	3.6	0.1	8.0
Maximum 8-hour average ozone (ppb)	9,039	43.0	44.8	3.2	102.0
Mean particulate matter, 10 µm or less (µg/m^3) [a]	8,331	39.0	38.5	8.0	158.0
Mean nitrogen dioxide (ppb) [a]	8,956	18.4	19.7	0.0	72.8

[a] Geometric means are provided. Values were natural-log-transformed in the regression analyses.

Table 3. Relative Risk of Preterm Birth and Percent of Preterm Births Attributable to 2-day Mean Apparent Temperature (AT) on a 24.9 °C Day vs. an 18.6 °C Day, Detroit, MI Area, May-September, 1991-2001.

Model	Knots in day-of-year spline	Covariates	Relative Risk (95% Confidence Interval)	Percent attributable (95% Confidence Interval)
1	2	none	1.11 (1.02, 1.09)	9.9 (2.4, 16.4)
2	5	none	1.11 (1.03, 1.20)	9.8 (2.6, 16.5)
3	8	none	1.09 (1.01, 1.18)	8.1 (1.0, 17.6)
4	5	solar radiation, wind speed, precipitation	1.12 (1.04, 1.21)	10.6 (3.8, 17.2)
5	5	solar radiation, wind speed, precipitation, inverse-odds weights[s]	1.12 (1.02, 1.21)	10.4 (2.2, 17.5)

[s]Inverse-odds weights calculated from the predicted odds of AT given lag day 0 and 1 of ozone, PM_{10} and NO_2.

Table 4. Relative Excess Risk Due to Interaction (RERI) for Interactions of 2-Day Mean Apparent Temperature (AT) on a 24.9 °C Day vs. an 18.6 °C Day with Maternal Characteristics, Detroit, MI Area, May-September, 1991-2001.

Characteristic	RERI	95% Confidence Interval
Black race	-1.5	-1.9, -1.0
Age 16-19 years	0.50	0.22, 0.76
Age > 30 years	0.34	0.08, 0.65
Low prenatal care	0.44	0.19, 0.65
No high school	0.19	-0.06, 0.43
Smoker	0.52	0.29, 0.76

1. With 5 knots in the day-of-year spline.

For peer review only

FIGURE LEGENDS

Figure 1. Directed Acyclic Graph of the Causal Framework for Mediation of the Association Between Apparent Temperature (AT) and Preterm Birth (PTB) by Air Pollutants.

Figure 2. Association Between Mean Apparent Temperature (AT) Over Lag Days 0-1 and Preterm Birth (PTB), modeling AT as a b-spline with 3 df.

For peer review only

For peer review only - http://bmjopen.bmj.com/site/about/guidelines.xhtml

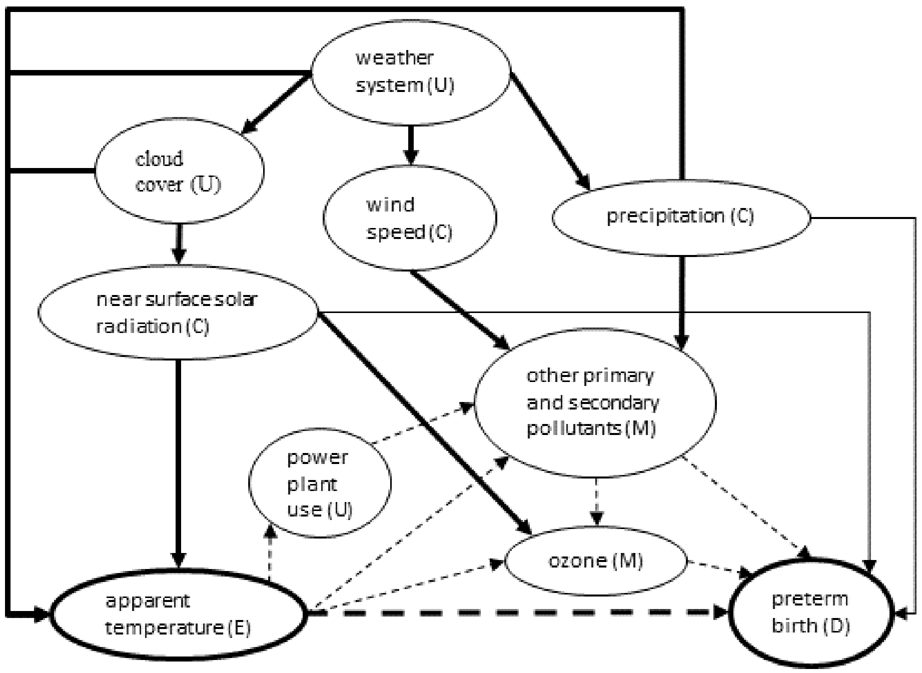

U = unmeasured, C = confounder, M = mediator, E = exposure of interest, D = outcome. Thick solid lines indicate known associations. Thin solid lines indicate suspected associations. The thick dashed line indicates the direct effect pathway being tested. The thin dashed lines indicate the indirect effect pathways being tested.
Other primary and secondary pollutants include particulate matter less than 10 microns in aerodynamic diameter and nitrogen dioxide.

Figure 1. Directed Acyclic Graph of the Causal Framework for Mediation of the Association Between Apparent Temperature (AT) and Preterm Birth (PTB) by Air Pollutants.

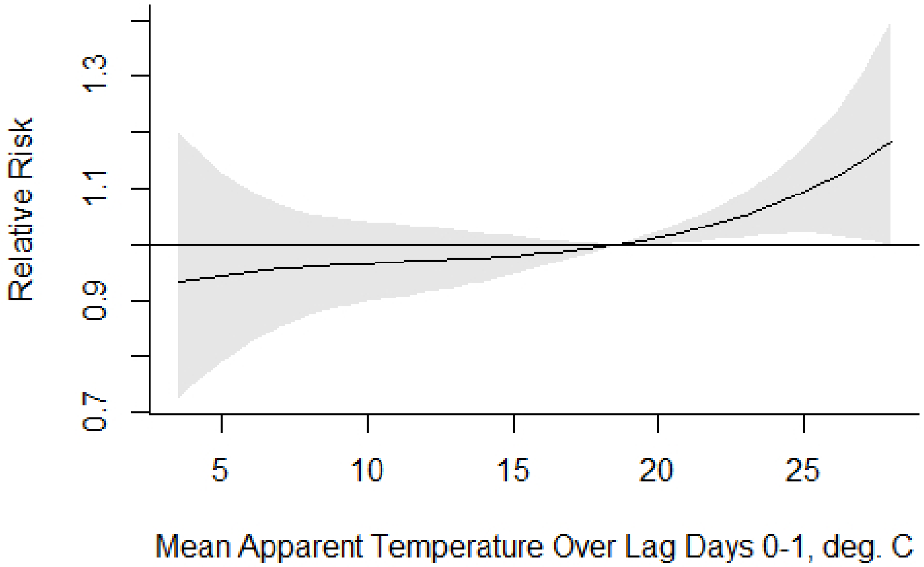

Figure 2. Association Between Mean Apparent Temperature (AT) Over Lag Days 0-1 and Preterm Birth (PTB), modeling AT as a b-spline with 3 df.

SUPPLEMENTAL MATERIAL

Table S1. Percent of preterm births attributable to 2-day mean apparent temperature (AT) on a 24.9 °C day vs. an 18.6 °C day, Detroit, MI, May-September, 1991-2001, using a case-crossover design.

Model	Time stratum	Covariates	Percent attributable	95% Confidence Interval
1	3 weeks	none	11.2	6.1, 15.9
2	2 weeks	none	11.7	6.9, 16.3
3	1 month	none	11.7	6.8, 16.4
4	3 weeks	solar radiation, wind speed, precipitation	18.7	1.3, 31.5
5	3 weeks	solar radiation, wind speed, precipitation, inverse-odds weights[s]	11.1	3.5, 17.5

[s]Inverse-odds weights calculated from the predicted odds of AT given lag day 0 and 1 of ozone, PM_{10} and NO_2.

For peer review only

A time series analysis of total and direct associations between high temperatures and preterm births in Detroit, Michigan

Journal:	*BMJ Open*
Manuscript ID	bmjopen-2019-032476.R2
Article Type:	Original research
Date Submitted by the Author:	17-Dec-2019
Complete List of Authors:	Gronlund, Carina; University of Michigan Institute for Social Research, Survey Research Center<br>Yang, Alyssa; Urban Indian Health Institute<br>Conlon, Kathryn; University of California Davis<br>Bergmans, Rachel S.; University of Michigan Medical School,<br>Le, Hien; Chemours Co<br>Batterman, Stuart; University of Michigan, Environmental Health Sciences<br>Wahl, Robert; Michigan Department of Health and Human Services<br>Cameron, Lorraine; Michigan Department of Health and Human Services<br>O'Neill, Marie; University of Michigan, Environmental Health Sciences and Epidemiology
**Primary Subject Heading**:	Epidemiology
Secondary Subject Heading:	Public health
Keywords:	Maternal medicine < OBSTETRICS, STATISTICS & RESEARCH METHODS, EPIDEMIOLOGY

SCHOLARONE™
Manuscripts

I, the Submitting Author has the right to grant and does grant on behalf of all authors of the Work (as defined in the below author licence), an exclusive licence and/or a non-exclusive licence for contributions from authors who are: i) UK Crown employees; ii) where BMJ has agreed a CC-BY licence shall apply, and/or iii) in accordance with the terms applicable for US Federal Government officers or employees acting as part of their official duties; on a worldwide, perpetual, irrevocable, royalty-free basis to BMJ Publishing Group Ltd ("BMJ") its licensees and where the relevant Journal is co-owned by BMJ to the co-owners of the Journal, to publish the Work in this journal and any other BMJ products and to exploit all rights, as set out in our licence.

The Submitting Author accepts and understands that any supply made under these terms is made by BMJ to the Submitting Author unless you are acting as an employee on behalf of your employer or a postgraduate student of an affiliated institution which is paying any applicable article publishing charge ("APC") for Open Access articles. Where the Submitting Author wishes to make the Work available on an Open Access basis (and intends to pay the relevant APC), the terms of reuse of such Open Access shall be governed by a Creative Commons licence – details of these licences and which Creative Commons licence will apply to this Work are set out in our licence referred to above.

Other than as permitted in any relevant BMJ Author's Self Archiving Policies, I confirm this Work has not been accepted for publication elsewhere, is not being considered for publication elsewhere and does not duplicate material already published. I confirm all authors consent to publication of this Work and authorise the granting of this licence.

For peer review only

TITLE PAGE

Title

A time series analysis of total and direct associations between high temperatures and preterm births in Detroit, Michigan

Authors

Carina J. Gronlund[1], Alyssa J. Yang[2], Kathryn C. Conlon[3], Rachel S. Bergmans[4], Hien Q. Le[5], Stuart A. Batterman[6], Robert L. Wahl[7], Lorraine Cameron[7], Marie S. O'Neill[6,8]

[1]Corresponding author:
University of Michigan Institute for Social Research
Survey Research Center, Social Environment and Health Program
426 Thompson St. Rm 3340
Ann Arbor, MI 48104
gronlund@umich.edu
734-615-9215

[2]Urban Indian Health Institute, Seattle, WA, USA

[3]University of California at Davis, Davis, CA, USA

[4]Department of Psychiatry, University of Michigan, Ann Arbor, MI, USA

[5]Health Services/Epidemiology, The Chemours Company, Wilmington, DE, USA

[6]Department of Environmental Health Sciences, University of Michigan, Ann Arbor, MI, USA

[7]Michigan Department of Health and Human Services, Lansing, MI, USA

[8]Department of Epidemiology, University of Michigan, Ann Arbor, MI, USA

Word count: 3,858

For peer review only

ABSTRACT

Objectives: Preterm births (PTBs) represent significant health risks, and several studies have found associations between high outdoor temperatures and PTB. We estimated both the total and natural direct effects (independent of particulate matter, ozone and nitrogen dioxide air pollutants) of the prior two-day mean apparent temperature (AT) on PTB. We evaluated effect modification by maternal age, race, education, smoking status, and prenatal care.

Design and Setting: We obtained birth records and meteorologic data for the Detroit, Michigan area for the warm months (May-September), 1991-2001. We used a time series Poisson regression with splines of AT, wind speed, solar radiation, and citywide average precipitation to estimate total effects. To accommodate multiple mediators and exposure-mediator interactions, AT inverse odds weights, predicted by meteorological and air pollutant covariates, were added in a subsequent model to estimate direct effects.

Results: At 24.9 °C relative to 18.6 °C, 10.6% (95% Confidence Interval: 3.8%, 17.2%) of PTBs were attributable to the total effects of AT, and 10.4% (95% CI: 2.2%, 17.5%) to direct effects. Relative excess risks of interaction indicated that the risk of PTB with increasing temperature above 18.6 °C was significantly lower among black mothers and higher among mothers less than 19, older than 30, with late or no prenatal care, and who smoked.

Conclusion: This additional evidence of a direct association between high temperature and PTB may motivate public health interventions to reduce extreme heat exposures among pregnant women, particularly among those who may have enhanced vulnerability.

For peer review only - http://bmjopen.bmj.com/site/about/guidelines.xhtml

STRENGTHS AND LIMITATIONS OF THIS STUDY

- We expand on a growing literature demonstrating total effects of temperature on PTB by examining mediation of the total temperature effect by air pollutants.
- We did not have geographic identifiers for the study participants at a level as fine as residential address or indoor exposure information, and we therefore assigned time-varying environmental exposures at an outdoor, citywide level.
- A low prevalence of residential air conditioning (32%) in the study time period suggests that our exposure assignment using outdoor exposures alone may have more accurately reflected total daily exposures than in more recent studies in other geographies.
- We account for multiple mediators and potential exposure-mediator interactions by applying a straightforward technique—the use of inverse odds weights—to separate the total effects of temperature on PTB from the direct effects.
- We used methods examining potential synergistic effects, as opposed to just examining statistical interactions, by other characteristics that confer vulnerability to PTB.

For peer review only

INTRODUCTION

Preterm births (PTBs) are defined as births that occur before 37 gestational weeks. Babies born prematurely are at greater risk of infant death and impaired health, such as cerebral palsy and impairments of cognitive development, hearing, vision, respiration, and digestion. In 2016, 10% of babies were born prematurely, with statewide rates ranging 7.8-13.7%[1]. Within-state disparities in preterm birth rates can be greater: from 1990-2010, 18% of births in Detroit, Michigan were PTBs[2].

Certain risk factors are known to be associated with PTB, such as cigarette smoking, alcohol use, hypertension, and diabetes[1]. Air pollution is associated with PTB[3 4], and higher ambient temperature has been proposed as a risk factor for PTB[5 6].

High ambient temperature could pose a risk for PTB through several biological pathways, including stress and dehydration pathways.[7] Several studies have evaluated possible associations between high ambient temperature and PTB, with mixed results[5 6]. Studies in cities in Australia, Quebec, China, Belgium, Italy, Spain and across the U.S. have all found significant positive associations between hot temperatures within the week preceding delivery and PTB[5 6 8-16]. High ambient temperatures in the month of conception and the third trimester were also positively associated with PTB in Changsha, China.[17,18] However, earlier, rigorously conducted time series analyses of temperature and PTB in Germany and in London, England did not find associations[19 20]. Likewise, in Chicago, Illinois, Porter et al. found no effect of the July 1995 heat wave on gestational length[21]. Sources of heterogeneity among these effect estimates may include differences in study design, prevailing climate and regional adaptation, population structure, exposure assessment, critical windows of exposure considered, and methods of gestational age assessment[12].

For peer review only - http://bmjopen.bmj.com/site/about/guidelines.xhtml

Studies often control for air pollution exposure, although the argument has been made that treating air pollutants as confounders of temperature-health associations is inappropriate given that high temperatures can contribute to increased concentrations of some air pollutants, and both temperature and air pollution are affected by sunlight[22]. Differences in the manner in which air pollutants are accounted for in the temperature-PTB modeling may also account for some of the heterogeneity observed in this literature.

We performed a time series analysis to investigate the association between high AT and PTB in the Detroit, Michigan area, estimating both the total effects of temperature as well as the natural direct effects. To estimate natural direct effects, we excluded potential mediation effects by the air pollutants ozone, particulate matter with an aerodynamic diameter of less than 10 micrometers (PM_{10}), and nitrogen dioxide (NO_2) using an inverse odds weighting technique. We further examined whether the maternal risk factors of age, race, education, smoking, and level of prenatal care modified the association between high AT and PTB.

METHODS

Outcome Variable

Birth outcome data were derived from an electronic database of birth certificate records requested from the Michigan Department of Health and Human Services Division for Vital Records and Health Statistics and subsequently limited to live, singleton births that occurred from January 1, 1991 to December 31, 2001 in ZIP codes within 4 km of one of three air quality monitors (Allen Park, Linwood, and East 7 Mile) in and bordering Detroit, Michigan[4]. In the final data set, births were categorized as term vs. PTB, defined as births that were less than 37 gestational weeks. We limited the data set to births that occurred May-September, in order to focus on heat exposures, for a total of 9,053 births

Identification of Confounders and Mediators

For peer review only - http://bmjopen.bmj.com/site/about/guidelines.xhtml

A directed acyclic graph (DAG) was constructed to define the causal framework and elucidate potential mediating effects of air pollutants in the association between AT and PTB (Figure 1). In this DAG, AT is the exposure of interest and PTB the outcome of interest. Given suspected associations between PTB and precipitation [23] and between PTB and vitamin D, for which one source is solar radiation [24], as well as causal meteorological relationships between AT, solar radiation, and precipitation via a parent weather system, precipitation and solar radiation are potential confounders of the AT-PTB association. In this DAG, primary and secondary air pollutants are the hypothesized mediators of the temperature-PTB association given that: 1) they have been associated with the health outcome in other locations[3] and in this dataset in the third trimester[4] , 2) high temperature enhances the rate of ground-level ozone formation, and 3) power plant emissions increase when temperatures rise to accommodate additional demand for air conditioning[25]. Furthermore, the weather system is a causal parent to both AT and the pollutants and therefore a potential confounder given that meteorological conditions such as wind speed, precipitation, and cloud cover can affect pollutant formation and exposure as well as sunlight, which increases AT and promotes ozone formation[26]. Therefore, a model characterizing the *total effect* of AT would need to include the confounders of solar radiation, wind speed, and precipitation but not include pollutants. A model characterizing the *direct effect* of AT, independent of air pollution mediation, would need to include these mediators. The modeling used to characterize direct and indirect effects is further described in the Statistical Analysis section below.

Exposure Variables

Daily mean temperature, dew point temperature, and wind speed were obtained from the National Center for Environmental Information Integrated Surface Daily Lite database of daily weather parameters from weather stations worldwide.[27] Data from the Detroit City Airport was

For peer review only - http://bmjopen.bmj.com/site/about/guidelines.xhtml

used due to its proximity to the mothers' ZIP codes of residence. To better represent thermal discomfort, we used apparent temperature (AT) rather than air temperature. AT is similar to heat index, and increases with both air temperature and relative humidity. AT was calculated using the following formula: [-2.653 + (0.994*Temperature in °C) + 0.0153*(Dew Point Temperature in °C)^2][28].[29] From Detroit City Airport, 11% and 15% of temperatures and dew points were missing, respectively, so data from Detroit Metropolitan Airport were used to replace these missing data. Among non-missing values, daily AT from Detroit City Airport was highly correlated with that from Detroit Metropolitan Airport (Pearson's correlation coefficient = 0.98).

The total amount of direct and diffuse solar radiation received on a horizontal surface during each 60-minute period at the Detroit City Airport was retrieved from the National Solar Radiation Database[30]. These data were modeled from meteorological data including cloud cover, aerosol, and ozone data from sources such as sun photometers, satellites and albedo data[31]. We further estimated daily means from the hourly solar radiation values.

Daily precipitation totals were obtained from Oregon State University's PRISM Climate Group[32]. These are modeled at a 4-km resolution based on observations and a climatologically-aided interpolation process. Rasters were cropped to the City of Detroit and daily citywide averages were calculated.

Daily eight-hour maximum ozone and daily mean NO_2 and PM_{10} concentrations were obtained from the Environmental Protection Agency for all Wayne County, Michigan monitors and averaged by day and pollutant[33]. Only daily monitor values for which at least a single daily or 18 of the 24-hourly values were available were retained, resulting in substantial missingness, which was addressed in the statistical analysis below.

For peer review only - http://bmjopen.bmj.com/site/about/guidelines.xhtml

Birth certificate data also included date of birth and maternal age group (16-19, 20-29, and over 30), race (black or white), smoking status (smoker vs. non-smoker), education level (less than high school vs. high school or higher), and level of prenatal care (prenatal care vs. late or no prenatal care), which were used in analyses of effect modification.

Ethics Review

The Michigan Department of Health and Human Services Institutional Review Board (study number 201302-03-XA) and the University of Michigan Institutional Review Board (study number HUM00071694) determined the study exempt from IRB review per Title 45 Code of Federal Regulations 46.101.(b)--research involving collection of existing data and information is recorded by the investigator in such a manner that subjects cannot be identified.

Patient and Public Involvement

Patients and the public were not involved in the design or planning of the study.

Statistical Analysis

Case-crossover analysis is commonly used in studies of PTB and temperature[12 13 34]. Because PTB is not a rare event in this particular population, the case-crossover odds ratios would not approximate risk ratios. Therefore, we used a time series design with Poisson regression, controlling for seasonal and long-term variations in PTB counts with a cubic b-spline for day-of-year, with 5, 2 or 8 knots, and a cubic b-spline for year with 2 knots. AT exposure was characterized as two-day mean AT, or the mean of the AT values on the day of and the day before birth. For AT and the covariates, non-linearity was considered by initially modeling each as a b-spline with three knots and selecting a single knot for subsequent modeling of the covariate as a piecewise linear spline when substantial nonlinearity was visually evident. The 95% confidence intervals (CIs) were constructed from the 2.5^{th} and 97.5^{th} percentiles of 500 bootstrapped samples. In sensitivity analyses, given its wide usage for this research question, a

For peer review only - http://bmjopen.bmj.com/site/about/guidelines.xhtml

time-stratified case-crossover design was used with time-strata defined as two- or three-week periods.

To account for missing air pollutant values, we conducted multiple imputations using chained equations. We used a more general model including lag days 0-2 of the above meteorological and pollutant values. The air pollutant values were well-predicted by lag days 0-2 meteorology and air pollution values, and from examinations of trace plots, or scatter plots of successive parameter estimates, we determined that a total of three imputations following two burn-ins was sufficient. All subsequent analyses were conducted on each of the three resulting data sets.

To estimate the total effects of AT, we included terms for solar radiation, wind speed, and precipitation in the Poisson model, which blocked all of the paths in the DAG from AT to PTB that did not pass through the mediators (Figure 1). However, to estimate natural direct effects, we used a more generalizable technique—inverse odds weighting—given that we had multiple mediators and as well as potential exposure-mediator interactions[35]. In this technique, we first fit a standard linear regression of two-day mean AT (meanAT01) on the air pollutant mediators and covariates (solar radiation, wind speed, precipitation). We then estimated:

$$\text{inverse odds} = 1 / \exp(\text{predicted meanAT01} / \sigma^2) \qquad \text{Equation 1}$$

where σ^2 was the model mean squared-error. The inverse odds were then used as weights in the subsequent analysis of PTB and AT with the other covariates but not the air pollutants. The weights render AT independent of the mediators, thereby allowing the estimation of AT separately from the effects of the air pollutant mediators on PTB. Total and direct effects were calculated from each of the three imputed data sets and then each averaged.

For peer review only - http://bmjopen.bmj.com/site/about/guidelines.xhtml

For peer review only

To assess the public health impact of AT on PTB, we estimated the attributable fraction (AF), or the percent of PTB attributable to high AT among women exposed to the high AT. The AF was estimated as: 1 / (1 – RR) * 100% where the RR was for the 95th percentile (24.9 °C) relative to the 50th percentile (18.6 °C) of May-September two-day mean AT. Indirect effects were estimated as the difference between the total effects and the direct effects. The 95% confidence intervals (CIs) were constructed from the 2.5th and 97.5th percentiles of 500 bootstrapped samples.

To understand how the effects varied among maternal subgroups, or effect modification of the PTB-AT association, we simultaneously included interaction terms between the AT spline and the indicator variables for black race, age 16-19, age 30 years or older, low prenatal care (late or no prenatal care), current smoker, and no high school education. We expanded the data set such that we had rows for each unique combination of date and daily exposures, race, age group, education, prenatal care, and smoking status. Given the large number of zero counts, we specified a negative binomial distribution rather than a Poisson distribution. For public health significance, we were interested in the absolute rather than the relative increase in PTB risk due to synergistic effects between temperature and each modifier of interest. Therefore we focused on a*additive*, rather than the*multiplicative*, interactions. Relative excess risk due to interaction (RERI)[36] was calculated for each potential modifier as:

$$RR_{AT=95th,EM=1} - RR_{AT=95th,EM=0} - RR_{AT=50th,EM=1} + 1 \quad \text{Equation 2}$$

where EM was the effect modifier of interest and the denominator of each RR was the risk at the 50th percentile of AT (18.6 °C) and the absence of the modifier (EM = 0). RERI confidence CIs were bootstrapped from 1,000 samples. Analyses were conducted in SAS 9.4 (Copyright 2016, SAS Institute Inc., Cary, NC, USA) using PROC GLIMMIX, which allows for multiple splines

For peer review only - http://bmjopen.bmj.com/site/about/guidelines.xhtml

in a Poisson regression. Figure 2 was constructed in R (R Foundation for Statistical Computing, Vienna, Austria) using the dlnm package[37] following modeling using the glm.nb function in the MASS package[38].

RESULTS

There were 9,053 singleton PTBs in this Detroit-area sample, May-September, 1991-2001 (Table 1). There were fewer PTBs in September compared to the other months, and consistent with Detroit's population decline, the number of PTBs declined with time. Considering individual characteristics, 27.0% of the PTBs were among white mothers and 71.1% were among black mothers. A majority of the mothers were non-smokers (73.1%), had prenatal care (55.6%), were 20-29 years of age (53.2%) and had at least a high school education (60.2%) (Table 1).

On average, AT exposure was 18.0 °C, and among the days when AT was greater than the time-period-specific daily median of 18.6 °C (i.e., for the upper end of the temperature spline), AT averaged 1.5 °C higher. A total of 53% of the cases occurred on days with no rain, so the geometric mean precipitation was 0.4 mm. The mean ozone concentration was 44.8 ppb and the maximum was 102 ppb (Table 2). In examining the daily time series of ozone over the study period, the three-year running averages of the fourth highest daily 8-hour maximum value of ozone ranged from 77 to 92 ppb, suggesting that the (current) National Ambient Air Quality Standards (70 ppb) had been exceeded in each year of the study.

In a crude model of the association between PTB and AT where AT was modeled flexibly as a b-spline with 3 df, we found a nonlinear association, with approximately null effects below 18-19 C and an increasingly stronger positive association at higher temperatures (Figure 2). To address potential sensitivity of the results to control for season, we varied the df in the

For peer review only - http://bmjopen.bmj.com/site/about/guidelines.xhtml

For peer review only

day-of-year term, using 2, 5, and 8 df. The results were not highly sensitive to this choice, with the point estimates of the percentages of PTB attributable to AT ranging only from 8.1% (8 df) to 9.9% (2 df) among pregnant women exposed to days when AT was 24.9 °C vs exposure on 18.6 °C days (Table 3). In case-crossover analyses, the resulting odds ratios were also statistically significantly greater than 1.0, regardless of whether the time strata were two-week, three-week, or one-month periods (Supplemental Material Table S1). However, the odds ratios were greater in magnitude than the risk ratios estimated in the time series design, which is expected, given that odds ratios overestimate risk ratios when events are not rare.

The total effects were estimated as 10.6% (95% CI: 3.8%, 17.2%) of PTBs attributable to AT among pregnant women exposed to a day when AT was 24.9 °C vs. exposure on an 18.6 °C day. Accounting for mediation of this effect by PM_{10}, ozone, and NO_2 by using inverse odds weights, the direct effect of AT on preterm birth was decreased to 10.4% (95% CI: 2.2%, 17.5%), although the difference between the two values was not statistically significant (indirect effect = 0.3%, 95% CI: -1.7%, 2.6%).

In examining the RERI from interactions between AT and maternal characteristics, black race was found to be protective, with an RERI significantly less than 0 (Table 4). The magnitudes of RERIs are not meaningful, but this result indicates that the association between high temperature and PTB is weaker among women of black race than non-black race. In contrast, RERIs for age 16-19 years, age > 30 years, low prenatal care and tobacco smoking were all significantly greater than 0, indicating that the association between PTB and high temperature is stronger among women with these characteristics.

DISCUSSION

For peer review only - http://bmjopen.bmj.com/site/about/guidelines.xhtml

The strong association between short-term temperature exposure and PTB in Detroit, Michigan was consistent with several other studies' observed associations between PTB or diminished gestational age and temperature exposures within the week prior to delivery at regionally high temperatures. Regions in which these associations have been found include Central Australia (8.3% at 40 °C daily maximum temperature)[11], Northern California, USA (11.6% increase for a 5.6 °C increase in weekly average AT in the warm season)[34], Alabama, USA (32.4% increase with two consecutive days of daily mean temperatures above the 98th percentile)[39], Barcelona, Spain (5-day reduction in average gestational age with heat index above the 99th percentile)[8], Rome, Italy (1.9% increase per 1 °C increase in maximum AT in the prior two days and 19% increase during heat waves)[40], Brisbane, Australia (13%-100% increase during heat waves), in an aggregated sample of 12 U.S. cities (12-16% increase with 2.8 °C increase in prior week)[12], in Southern China (7% increase with previous-week temperatures above 95th percentile)[10], and in a multi-city USA sample (2% increased PTB risk with extreme heat in the prior 4 days) [41].

The precise biological mechanisms by which high ambient temperature might increase risk for PTB are unclear, although psychosocial stress[7] and dehydration pathways are plausible.[7]. Stress increases the levels of cortisol and epinephrine, potentially leading to the secretion of placental corticotropin-releasing hormones (CRH). Placental CRH can then activate the fetal hypothalamic-pituitary-adrenal (HPA) axis, which could prompt the fetal expression of cortisol and dehydroepiandrosterone-sulfate (DHEA-S) and placental release of estriol and prostaglandins, potentially triggering PTB[42]. Dehydration due to high temperatures and sweating could also reduce blood flow to the uterus and induce a greater release of antidiuretic hormone and oxytocin, which could trigger labor onset[43].

For peer review only - http://bmjopen.bmj.com/site/about/guidelines.xhtml

One strength of our study may be a tighter correspondence between the true ambient temperatures experienced by our sample and the measured outdoor ambient AT given the low air conditioning prevalence in the Detroit area during the time period. Specifically, in 1993, the last year for which county-specific American Housing Survey data were available in the Detroit area, only 32% of Wayne County households had central air conditioning[44]. Furthermore, central air conditioning prevalence was low in this region despite a "hot-summer humid continental climate" Köppen Climate Classification[45], allowing a fairly wide range of warm season AT exposure over which AT-PTB associations could be examined. In contrast, regions where associations between temperature and PTB were null in rigorously conducted daily time series studies included Brandenburg and Saxony, Germany[20] and London, UK[19], which are in "temperate oceanic climates," where all months have average temperatures below 22 °C [45]. Additionally, Guo et al.[10] found an association between previous-week temperature and PTB only in the "hot" region of China, defined as having annual average temperatures 19 °C or higher. Furthermore, in a survival analysis of previous-week temperature and PTB in 18 European cities, the pooled effect estimates were null, and no individual city results were presented[46]. Again, with the exception of 4 cities, these European cities were all in cooler climates where all months have average temperatures below 22 °C[45]. This suggests that in regions where the threat of extremely high temperatures is rare, PTB is not triggered at warm temperatures, regardless of relative temperature thresholds, even at the same absolute temperatures at which heat-associated PTB is experienced in warmer climates. This could be due to differences in emotional stress[47] or physiological stress responses to warm temperatures between climates. Alternatively, the heterogeneity in effect estimates could be due to regional differences in the misclassification of the true individually-experienced temperatures by outdoor

For peer review only - http://bmjopen.bmj.com/site/about/guidelines.xhtml

temperatures. Finally, in the 18-city European study, the PTB rate was only 5%, suggesting that PTB etiologies may differ between Europe and the U.S., in which case the pregnancies in the European cohorts could have been less susceptible physiologically to high temperature[46].Other strengths of our study include our consideration of near-surface solar radiation and weather conditions as confounders of the total effects of AT, air pollutants as mediators rather than confounders, and estimation of the direct effects of AT as distinct from those mediated by air pollutants. By using an analysis technique that was "agnostic" to exposure-mediator interactions and could accommodate multiple mediators[35], we found that most if not all of the effect of AT on PTB is direct, and not mediated by daily changes in air pollution concentrations. However, this mediation analysis technique tends to over-estimate the confidence intervals around the indirect effects[35], which may account for our finding a null indirect effect of air pollutants on PTB when previous research has in fact identified significant associations between short-term increases in air pollution and PTB[48]. We may have also underestimated the air pollutant effects because we were only examining the indirect effects of AT through an air pollutant pathway rather than the total effects of air pollution and because we only considered PM_{10} rather than $PM_{2.5}$.

Another strength of our study is that we reported RERIs, rather than merely reporting statistical interactions in the models. In doing so, we provided evidence for synergistic effects of high temperature with the independent risk factors for PTB of prenatal care, smoking status, and age. Interestingly, in this majority-black population, the risk of PTB with high temperatures was actually lower among black mothers, after controlling for age, education, smoking status, and prenatal care.

Limitations include the fact that we were not able to distinguish between spontaneous and medically indicated PTBs. Considering that medically indicated preterm deliveries are

For peer review only - http://bmjopen.bmj.com/site/about/guidelines.xhtml

unlikely to be related to temperature, this limitation affects the generalizability of our relative risks to populations where the relative percentages of spontaneous vs. medically indicated PTB differ. We also did not have information on how much earlier the birth was, e.g., a one-day premature birth vs. a six-week premature birth. This prevented us from including an offset for the population of pregnancies at-risk of PTB[49]. Our model included a spline for year and spline for day-of-year to attempt to capture within-season variations in PTBs. Varying the degrees of freedom in the day-of-year term from 2 to 8 had only a mild effect on the relative risks, although this would not have captured sub-weekly changes in PTBs. Another limitation is our dependence on last menstrual period rather than ultrasound-derived gestational age. Last menstrual period tends to result in more births being classified as preterm, particularly among African Americans[50]. This limitation, assuming it was not correlated with temperature, would bias our results towards the null.

An additional limitation is that the prevalence of characteristics enhancing vulnerability to PTB may have changed since the study period, thereby decreasing the generalizability of these results to the present-day population of this region. Future research should employ present-day cohorts of pregnant women, for which a denominator of total pregnancies is therefore available, linked with refined temperature exposure measurements, including indoor and neighborhood temperature exposure estimates. These research refinements will better characterize the thermal exposures and severity of heat-induced PTB, or more specifically, gestational length, and better identify which pregnancies are particularly vulnerable to early parturition on hot days.

Despite the aforementioned limitations, given the evidence from this and other studies, pregnant women, in addition to older adults, should be considered as a group vulnerable to short-

For peer review only - http://bmjopen.bmj.com/site/about/guidelines.xhtml

term heat health effects when considering housing and climate adaptation measures in Detroit and similar or warmer climates.

For peer review only

For peer review only - http://bmjopen.bmj.com/site/about/guidelines.xhtml

CONTRIBUTORSHIP STATEMENT

MSO, SAB, HQL, RLW, and LC obtained the data or resources, critiqued the analysis, and reviewed the manuscript. RSB and KCC assisted with preliminary analyses, critiqued the analysis and reviewed the manuscript. AJY performed and drafted preliminary analyses and reviewed the manuscript. CJG directed preliminary analyses, revised the analysis, and revised the manuscript. We thank Leah Comment for early-stage data management and analysis assistance. We also thank Patricia Maina for her contribution to the preterm birth and heat literature review and Sung Kyun Park for early stage advice.

FUNDING

This work was supported by a Michigan Institute for Clinical and Health Research Postdoctoral Translational Scholars Fellowship (2UL1TR000433-06), National Institute of Environmental Health Sciences grants K99ES026198 and P30ES017885, Cooperative Agreement Number EH001124 from the Centers for Disease Control and Prevention (CDC), National Science Foundation grant 1520803, and CDC/ National Institute for Occupational Safety and Health grant T42 OH008455. Its contents are solely the responsibility of the authors and do not necessarily represent the official views of the CDC or the Michigan Department of Health and Human Services. None of the funders participated in the design, collection, analysis, or interpretation of the data.

For peer review only

COMPETING INTERESTS

None declared.

DATA SHARING STATEMENT

Birth outcome data were derived from birth certificate records kept by the Michigan Department of Health and Human Services (MDHHS).[4] These confidential data may be obtained from the MDHHS Division for Vital Records and Health Statistics following completion of a data use agreement and IRB approval. The exposure data are publicly available and were obtained as described in the Methods. The processed exposure data may be requested from C. Gronlund (gronlund@umich.edu).

For peer review only - http://bmjopen.bmj.com/site/about/guidelines.xhtml

REFERENCES

1. Centers for Disease Control and Prevention (CDC). Premature Birth 2017 [updated 11/6/2017. Available from: https://www.cdc.gov/features/prematurebirth/ accessed Jan 29 2018.
2. Make Your Date Detroit, Center for Advanced Obstetrical Care and Research, Wayne State University. Why This Matters--Facts 2017 [Available from: https://makeyourdate.org/facts/ accessed Jan 29 2018.
3. Sun XL, Luo XP, Zhao CM, et al. The association between fine particulate matter exposure during pregnancy and preterm birth: a meta-analysis. *BMC Pregnancy Childbirth* 2015;15:12. doi: 10.1186/s12884-015-0738-2
4. Le HQ, Batterman SA, Wirth JJ, et al. Air pollutant exposure and preterm and term small-for-gestational-age births in Detroit, Michigan: Long-term trends and associations. *Environment International* 2012;44:7-17. doi: http://dx.doi.org/10.1016/j.envint.2012.01.003
5. Carolan-Olah M, Frankowska D. High environmental temperature and preterm birth: a review of the evidence. *Midwifery* 2014;30(1):50-9. doi: 10.1016/j.midw.2013.01.011 [published Online First: 2013/03/12]
6. Strand LB, Barnett AG, Tong S. The influence of season and ambient temperature on birth outcomes: a review of the epidemiological literature. *Environ Res* 2011;111(3):451-62. doi: 10.1016/j.envres.2011.01.023 [published Online First: 2011/02/22]
7. McMorris T, Swain J, Smith M, et al. Heat stress, plasma concentrations of adrenaline, noradrenaline, 5-hydroxytryptamine and cortisol, mood state and cognitive performance. *International Journal of Psychophysiology* 2006;61(2):204-15. doi: https://doi.org/10.1016/j.ijpsycho.2005.10.002
8. Dadvand P, Basagana X, Sartini C, et al. Climate extremes and the length of gestation. *Environ Health Perspect* 2011;119(10):1449-53. doi: 10.1289/ehp.1003241 [published Online First: 2011/06/11]
9. Strand LB, Barnett AG, Tong S. Maternal exposure to ambient temperature and the risks of preterm birth and stillbirth in Brisbane, Australia. *Am J Epidemiol* 2012;175(2):99-107. doi: 10.1093/aje/kwr404 [published Online First: 2011/12/15]
10. Guo TJ, Wang YY, Zhang HG, et al. The association between ambient temperature and the risk of preterm birth in China. *Sci Total Environ* 2018;613:439-46. doi: 10.1016/j.scitotenv.2017.09.104
11. Mathew S, Mathur D, Chang AB, et al. Examining the Effects of Ambient Temperature on Pre-Term Birth in Central Australia. *International Journal of Environmental Research and Public Health* 2017;14(2) doi: 10.3390/ijerph14020147
12. Ha SD, Liu DP, Zhu YY, et al. Ambient Temperature and Early Delivery of Singleton Pregnancies. *Environmental Health Perspectives* 2017;125(3):453-59. doi: 10.1289/ehp97
13. Basu R, Chen H, Li DK, et al. The impact of maternal factors on the association between temperature and preterm delivery. *Environmental Research* 2017;154:109-14. doi: 10.1016/j.envres.2016.12.017
14. Schifano P, Asta F, Dadvand P, et al. Heat and air pollution exposure as triggers of delivery: A survival analysis of population-based pregnancy cohorts in Rome and Barcelona. *Environment International* 2016;88:153-59. doi: 10.1016/j.envint.2015.12.013

For peer review only - http://bmjopen.bmj.com/site/about/guidelines.xhtml

15. He JR, Liu Y, Xia XY, et al. Ambient Temperature and the Risk of Preterm Birth in Guangzhou, China (2001-2011). *Environ Health Perspect* 2015 doi: 10.1289/ehp.1509778 [published Online First: 2015/12/17]
16. Arroyo V, Diaz J, Ortiz C, et al. Short term effect of air pollution, noise and heat waves on preterm births in Madrid (Spain). *Environmental Research* 2016;145:162-68. doi: 10.1016/j.envres.2015.11.034
17. Zheng XR, Zhang WS, Lu C, et al. An epidemiological assessment of the effect of ambient temperature on the incidence of preterm births: Identifying windows of susceptibility during pregnancy. *Journal of Thermal Biology* 2018;74:201-07. doi: 10.1016/j.jtherbio.2018.04.001
18. Zhong Q, Lu C, Zhang WS, et al. Preterm birth and ambient temperature: Strong association during night-time and warm seasons. *Journal of Thermal Biology* 2018;78:381-90. doi: 10.1016/j.jtherbio.2018.11.002
19. Lee SJ, Hajat S, Steer PJ, et al. A time-series analysis of any short-term effects of meteorological and air pollution factors on preterm births in London, UK. *Environ Res* 2008;106(2):185-94. doi: 10.1016/j.envres.2007.10.003 [published Online First: 2007/11/21]
20. Wolf J, Armstrong B. The association of season and temperature with adverse pregnancy outcome in two German states, a time-series analysis. *PLoS One* 2012;7(7):e40228. doi: 10.1371/journal.pone.0040228 [published Online First: 2012/07/14]
21. Porter KR, Thomas SD, Whitman S. The relation of gestation length to short-term heat stress. *American Journal of Public Health* 1999;89(7):1090-92. doi: 10.2105/ajph.89.7.1090
22. Buckley JP, Samet JM, Richardson DB. Commentary: Does air pollution confound studies of temperature? *Epidemiology* 2014;25(2):242-5. doi: 10.1097/ede.0000000000000051 [published Online First: 2014/02/04]
23. Yu X, Feric Z, Cordero JF, et al. Potential influence of temperature and precipitation on preterm birth rate in Puerto Rico. *Scientific reports* 2018;8:9. doi: 10.1038/s41598-018-34179-z
24. Dovnik A, Mujezinović F. The Association of Vitamin D Levels with Common Pregnancy Complications. *Nutrients* 2018;10(7):867. doi: 10.3390/nu10070867
25. Abel D, Holloway T, Kladar RM, et al. Response of Power Plant Emissions to Ambient Temperature in the Eastern United States. *Environ Sci Technol* 2017;51(10):5838-46. doi: 10.1021/acs.est.6b06201
26. Environmental Protection Agency (EPA). Ozone Pollution and Your Patient's Health: What is Ozone? 2018 [Available from: https://www.epa.gov/ozone-pollution-and-your-patients-health/what-ozone accessed December 20 2018.
27. National Climatic Data Center. Integrated Surface Database Lite 2010 [Available from: http://www.ncdc.noaa.gov/oa/climate/isd/index.php accessed July 2010.
28. Kalkstein LS, Valimont, K.M. An evaluation of summer discomfort in the United States using a relative climatological index. *Bull Am Meteorol Soc* 1986;7:842-48.
29. Oswald EM, Rood RB, Zhang K, et al. An investigation into the spatial variability of near-surface air temperatures in the Detroit, MI metropolitan region. *J Appl Meteorol Climatol* 2012;51(7):1290-304. doi: 10.1175/JAMC-D-11-0127.1
30. National Solar Radiation Database, 1991-2010 Update [Available from: https://rredc.nrel.gov/solar/old_data/nsrdb/ accessed August 25 2017.

For peer review only - http://bmjopen.bmj.com/site/about/guidelines.xhtml

31. Wilcox S. National Solar Radiation Database 1991-2010 Update: User's Manual. In: National Renewable Energy Laboratory, ed.: U.S. Department of Energy, Office of Energy Efficiency & Renewable Energy, 2012.
32. PRISM Climate Group. PRISM Climate Data 2019 [Available from: http://www.prism.oregonstate.edu/ accessed March 6 2016.
33. U.S. Environmental Protection Agency. AirData 2015 [Available from: http://aqsdr1.epa.gov/aqsweb/aqstmp/airdata/download_files.html accessed Feb 5 2015.
34. Avalos LA, Chen H, Li DK, et al. The impact of high apparent temperature on spontaneous preterm delivery: a case-crossover study. *Environmental Health* 2017;16 doi: 10.1186/s12940-017-0209-5
35. Nguyen QC, Osypuk TL, Schmidt NM, et al. Practical Guidance for Conducting Mediation Analysis With Multiple Mediators Using Inverse Odds Ratio Weighting. *American Journal of Epidemiology* 2015;181(5):349-56. doi: 10.1093/aje/kwu278
36. Nie L, Chu H, Li F, et al. Relative Excess Risk Due to Interaction: Resampling-based Confidence Intervals. *Epidemiology* 2010;21(4):552-56.
37. Gasparrini A, Armstrong B, Kenward MG. Distributed lag non-linear models. *Stat Med* 2010;29(21):2224-34. doi: 10.1002/sim.3940 [doi] [published Online First: 2010/09/03]
38. Venables WN, Ripley BD. Functions and Datasets to Support "Modern Applied Statistics with S" (4th edition, 2002) 2018 [Available from: https://cran.r-project.org/web/packages/MASS/index.html accessed April 1 2019.
39. Kent ST, McClure LA, Zaitchik BF, et al. Heat Waves and Health Outcomes in Alabama (USA): The Importance of Heat Wave Definition. *Environ Health Perspect* 2013 doi: 10.1289/ehp.1307262 [published Online First: 2013/11/26]
40. Schifano P, Lallo A, Asta F, et al. Effect of ambient temperature and air pollutants on the risk of preterm birth, Rome 2001-2010. *Environ Int* 2013;61:77-87. doi: 10.1016/j.envint.2013.09.005 [published Online First: 2013/10/10]
41. Sun SZ, Weinberger KR, Spangler KR, et al. Ambient temperature and preterm birth: A retrospective study of 32 million US singleton births. *Environment International* 2019;126:7-13. doi: 10.1016/j.envint.2019.02.023
42. Behrman RE, Adashi EY, Allen MC, et al. Preterm Birth: Causes, Consequences, and Prevention. In: Medicine Io, ed. Report Brief. Washington, DC: Institute of Medicine, 2006.
43. Stan C, Boulvain M, Hirsbrunner-Amagbaly P, et al. Hydration for treatment of preterm labour. *Cochrane Database Syst Rev* 2002;2
44. United States. Bureau of the Census. American Housing Survey, 1993: MSA Core and Supplement File: [distributor], 2006.
45. Peel MC, Finlayson BL, McMahon TA. Updated world map of the Köppen-Geiger climate classification. *Hydrol Earth Syst Sci* 2007;11(5):1633-44. doi: 10.5194/hess-11-1633-2007
46. Giorgis-Allemand L, Pedersen M, Bernard C, et al. The Influence of Meteorological Factors and Atmospheric Pollutants on the Risk of Preterm Birth. *American Journal of Epidemiology* 2017;185(4):247-58. doi: 10.1093/aje/kww141
47. Lin YF, Hu WJ, Xu J, et al. Association between temperature and maternal stress during pregnancy. *Environmental Research* 2017;158:421-30. doi: 10.1016/j.envres.2017.06.034

For peer review only - http://bmjopen.bmj.com/site/about/guidelines.xhtml

48. Stieb DM, Lavigne E, Chen L, et al. Air pollution in the week prior to delivery and preterm birth in 24 Canadian cities: a time to event analysis. *Environ Health* 2019;18(1):1. doi: 10.1186/s12940-018-0440-8 [published Online First: 2019/01/05]
49. Vicedo-Cabrera AM, Iniguez C, Barona C, et al. Exposure to elevated temperatures and risk of preterm birth in Valencia, Spain. *Environmental Research* 2014;134:210-17. doi: 10.1016/j.envres.2014.07.021
50. Wingate MS, Alexander GR, Buekens P, et al. Comparison of Gestational Age Classifications: Date of Last Menstrual Period vs. Clinical Estimate. *Annals of epidemiology* 2007;17(6):425-30. doi: https://doi.org/10.1016/j.annepidem.2007.01.035

For peer review only

For peer review only - http://bmjopen.bmj.com/site/about/guidelines.xhtml

TABLES

Table 1. Demographics of Preterm Births in Detroit, MI Study Area, May-September, 1991-2001.

	N	%
Total	9,053	100
Month		
May	1,918	21.2
June	1,944	21.5
July	1,903	21.0
August	1.754	19.4
September	1,534	16.9
Year (Two Equal Time Periods)		
1991-1995	4,708	56.2
1996-2000	3,670	43.8
Maternal Race		
White	2,443	27.0
Black	6,433	71.1
Other	177	1.9
Maternal Smoking Status		
Non-Smoker	6,621	73.1
Smoker	2,345	25.9
Missing	87	1.0
Level of Prenatal Care		
Prenatal Care	5,037	55.6
Late or No Prenatal Care	2,960	32.7
Missing	1,056	11.7
Maternal Age		
16-19 years	1,782	19.7
20-29 years	4,821	53.2
≥ 30 years	2,450	27.1
Maternal Education		
Less than High School	3,485	38.5
High School or Higher	5,451	60.2
Missing	117	1.3

For peer review only - http://bmjopen.bmj.com/site/about/guidelines.xhtml

Table 2. Daily Exposures Among the PTB Cases, Detroit, MI Area, May-September, 1991-2001.

	N	Median	Mean	Min	Max
Two-day mean apparent temperature, °C	9,053	18.6	18.0	3.1	28.0
Two-day mean apparent temperature 18.6 °C and above	9,053	0.1	1.5	0.0	9.4
Mean solar radiation (W/m^2)	9,053	226.4	217.7	38.1	352.8
Total precipitation (mm)[a]	9,053	0.0	0.4	0.0	56.7
Mean wind speed (m/s)	9,053	3.6	3.6	0.1	8.0
Maximum 8-hour average ozone (ppb)	9,039	43.0	44.8	3.2	102.0
Mean particulate matter, 10 µm or less ($\mu g/m^3$) [a]	8,331	39.0	38.5	8.0	158.0
Mean nitrogen dioxide (ppb) [a]	8,956	18.4	19.7	0.0	72.8

[a] Geometric means are provided. Values were natural-log-transformed in the regression analyses.

Table 3. Relative Risk of Preterm Birth and Percent of Preterm Births Attributable to 2-day Mean Apparent Temperature (AT) on a 24.9 °C Day vs. an 18.6 °C Day, Detroit, MI Area, May-September, 1991-2001.

Model	Knots in day-of-year spline	Covariates	Relative Risk (95% Confidence Interval)	Percent attributable (95% Confidence Interval)
1	2	none	1.11 (1.02, 1.09)	9.9 (2.4, 16.4)
2	5	none	1.11 (1.03, 1.20)	9.8 (2.6, 16.5)
3	8	none	1.09 (1.01, 1.18)	8.1 (1.0, 17.6)
4	5	solar radiation, wind speed, precipitation	1.12 (1.04, 1.21)	10.6 (3.8, 17.2)
5	5	solar radiation, wind speed, precipitation, inverse-odds weights[s]	1.12 (1.02, 1.21)	10.4 (2.2, 17.5)

[s]Inverse-odds weights calculated from the predicted odds of AT given lag day 0 and 1 of ozone, PM_{10} and NO_2.

Table 4. Relative Excess Risk Due to Interaction (RERI) for Interactions of 2-Day Mean Apparent Temperature (AT) on a 24.9 °C Day vs. an 18.6 °C Day with Maternal Characteristics, Detroit, MI Area, May-September, 1991-2001.

Characteristic	RERI	95% Confidence Interval
Black race	-1.5	-1.9, -1.0
Age 16-19 years	0.50	0.22, 0.76
Age > 30 years	0.34	0.08, 0.65
Low prenatal care	0.44	0.19, 0.65
No high school	0.19	-0.06, 0.43
Smoker	0.52	0.29, 0.76

1. With 5 knots in the day-of-year spline.

For peer review only - http://bmjopen.bmj.com/site/about/guidelines.xhtml

FIGURE LEGENDS

Figure 1. Directed Acyclic Graph of the Causal Framework for Mediation of the Association Between Apparent Temperature (AT) and Preterm Birth (PTB) by Air Pollutants.

Figure 2. Association Between Mean Apparent Temperature (AT) Over Lag Days 0-1 and Preterm Birth (PTB), modeling AT as a b-spline with 3 df.

For peer review only

For peer review only - http://bmjopen.bmj.com/site/about/guidelines.xhtml

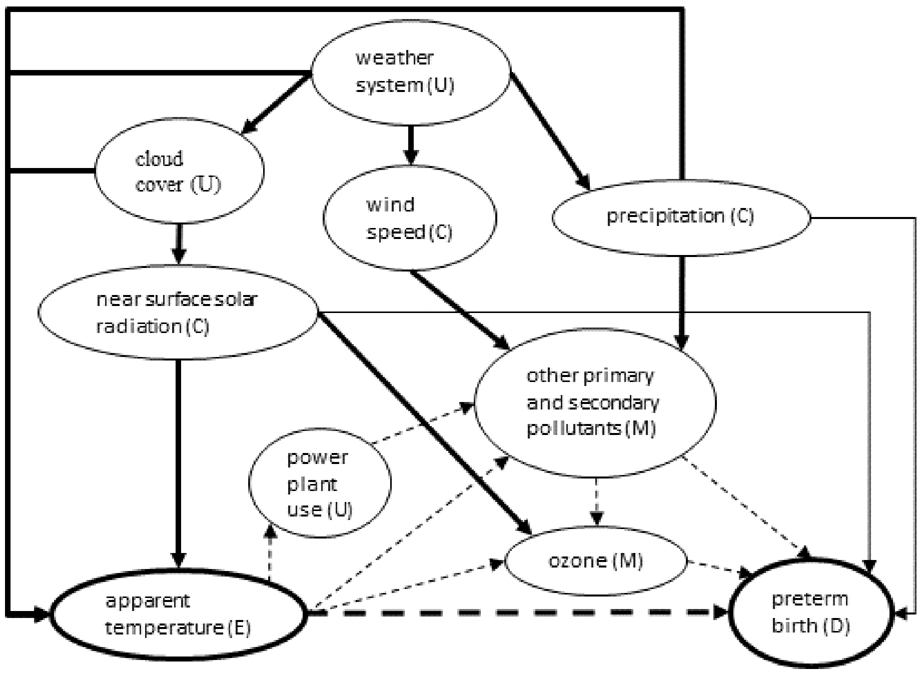

U = unmeasured, C = confounder, M = mediator, E = exposure of interest, D = outcome. Thick solid lines indicate known associations. Thin solid lines indicate suspected associations. The thick dashed line indicates the direct effect pathway being tested. The thin dashed lines indicate the indirect effect pathways being tested.
Other primary and secondary pollutants include particulate matter less than 10 microns in aerodynamic diameter and nitrogen dioxide.

Figure 1. Directed Acyclic Graph of the Causal Framework for Mediation of the Association Between Apparent Temperature (AT) and Preterm Birth (PTB) by Air Pollutants.

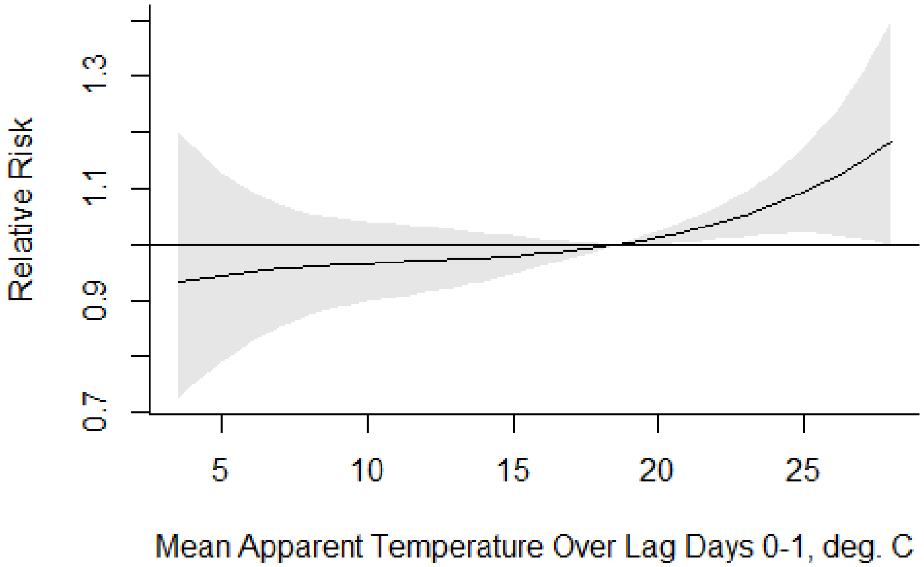

Figure 2. Association Between Mean Apparent Temperature (AT) Over Lag Days 0-1 and Preterm Birth (PTB), modeling AT as a b-spline with 3 df.

SUPPLEMENTAL MATERIAL

Table S1. Percent of preterm births attributable to 2-day mean apparent temperature (AT) on a 24.9 °C day vs. an 18.6 °C day, Detroit, MI, May-September, 1991-2001, using a case-crossover design.

Model	Time stratum	Covariates	Percent attributable	95% Confidence Interval
1	3 weeks	none	11.2	6.1, 15.9
2	2 weeks	none	11.7	6.9, 16.3
3	1 month	none	11.7	6.8, 16.4
4	3 weeks	solar radiation, wind speed, precipitation	18.7	1.3, 31.5
5	3 weeks	solar radiation, wind speed, precipitation, inverse-odds weights[s]	11.1	3.5, 17.5

[s]Inverse-odds weights calculated from the predicted odds of AT given lag day 0 and 1 of ozone, PM_{10} and NO_2.

For peer review only

STROBE 2007 (v4) checklist of items to be included in reports of observational studies in epidemiology*

Checklist for cohort, case-control, and cross-sectional studies (combined)

Section/Topic	Item #	Recommendation	Reported on page #
**Title and abstract**	1	(*a*) Indicate the study's design with a commonly used term in the title or the abstract	1
		(*b*) Provide in the abstract an informative and balanced summary of what was done and what was found	2
**Introduction**			
Background/rationale	2	Explain the scientific background and rationale for the investigation being reported	4-5
Objectives	3	State specific objectives, including any pre-specified hypotheses	5
**Methods**			
Study design	4	Present key elements of study design early in the paper	5
Setting	5	Describe the setting, locations, and relevant dates, including periods of recruitment, exposure, follow-up, and data collection	5-8
Participants	6	(*a*) *Cohort study*—Give the eligibility criteria, and the sources and methods of selection of participants. Describe methods of follow-up<br>*Case-control study*—Give the eligibility criteria, and the sources and methods of case ascertainment and control selection. Give the rationale for the choice of cases and controls<br>*Cross-sectional study*—Give the eligibility criteria, and the sources and methods of selection of participants	5-6
		(*b*) *Cohort study*—For matched studies, give matching criteria and number of exposed and unexposed<br>*Case-control study*—For matched studies, give matching criteria and the number of controls per case	5-6, Table 1
Variables	7	Clearly define all outcomes, exposures, predictors, potential confounders, and effect modifiers. Give diagnostic criteria, if applicable	5-8, Tables 1-2
Data sources/ measurement	8*	For each variable of interest, give sources of data and details of methods of assessment (measurement). Describe comparability of assessment methods if there is more than one group	*5-8, Tables 1-2*
Bias	9	Describe any efforts to address potential sources of bias	5-8
Study size	10	Explain how the study size was arrived at	5
Quantitative variables	11	Explain how quantitative variables were handled in the analyses. If applicable, describe which groupings were chosen and why	5-8
Statistical methods	12	(*a*) Describe all statistical methods, including those used to control for confounding	8-11
		(*b*) Describe any methods used to examine subgroups and interactions	10
		(*c*) Explain how missing data were addressed	9
		(*d*) *Cohort study*—If applicable, explain how loss to follow-up was addressed<br>*Case-control study*—If applicable, explain how matching of cases and controls was addressed	8-9

For peer review only

		Cross-sectional study—If applicable, describe analytical methods taking account of sampling strategy	
		(*e*) Describe any sensitivity analyses	9
**Results**			
Participants	13*	(a) Report numbers of individuals at each stage of study—eg numbers potentially eligible, examined for eligibility, confirmed eligible, included in the study, completing follow-up, and analysed	Table 1
		(b) Give reasons for non-participation at each stage	NA
		(c) Consider use of a flow diagram	NA
Descriptive data	14*	(a) Give characteristics of study participants (eg demographic, clinical, social) and information on exposures and potential confounders	Tables 1-2 and text
		(b) Indicate number of participants with missing data for each variable of interest	Table 1
		(c) *Cohort study*—Summarise follow-up time (eg, average and total amount)	NA
Outcome data	15*	*Cohort study*—Report numbers of outcome events or summary measures over time	NA
		Case-control study—Report numbers in each exposure category, or summary measures of exposure	Table 2 and text
		Cross-sectional study—Report numbers of outcome events or summary measures	NA
Main results	16	(*a*) Give unadjusted estimates and, if applicable, confounder-adjusted estimates and their precision (eg, 95% confidence interval). Make clear which confounders were adjusted for and why they were included	Table 3 and text
		(*b*) Report category boundaries when continuous variables were categorized	Table 1
		(*c*) If relevant, consider translating estimates of relative risk into absolute risk for a meaningful time period	Table 3 and text
Other analyses	17	Report other analyses done—eg analyses of subgroups and interactions, and sensitivity analyses	Tables 3-4 and text
**Discussion**			
Key results	18	Summarise key results with reference to study objectives	13
Limitations	19	Discuss limitations of the study, taking into account sources of potential bias or imprecision. Discuss both direction and magnitude of any potential bias	14-16
Interpretation	20	Give a cautious overall interpretation of results considering objectives, limitations, multiplicity of analyses, results from similar studies, and other relevant evidence	13, 18
Generalisability	21	Discuss the generalisability (external validity) of the study results	13-15, 18
**Other information**			
Funding	22	Give the source of funding and the role of the funders for the present study and, if applicable, for the original study on which the present article is based	18

*Give information separately for cases and controls in case-control studies and, if applicable, for exposed and unexposed groups in cohort and cross-sectional studies.
Note: An Explanation and Elaboration article discusses each checklist item and gives methodological background and published examples of transparent reporting. The STROBE checklist is best used in conjunction with this article (freely available on the Web sites of PLoS Medicine at http://www.plosmedicine.org/, Annals of Internal Medicine at http://www.annals.org/, and Epidemiology at http://www.epidem.com/). Information on the STROBE Initiative is available at www.strobe-statement.org.

For peer review only
